# Quinolone Antimicrobials: A Medicinal Chemistry Perspective on Structural Evolution, Pharmacology, and Computational Profiling

**DOI:** 10.3390/molecules31213949

**Published:** 2026-11-02

**Authors:** Gustavo Machado das Neves, Graziela de Araújo Lock, Itamar Luís Gonçalves, Teresa Cristina Tavares Dalla Costa, Vera Lucia Eifler-Lima

**Affiliations:** 1 Medicinal Organic Synthesis Laboratory (LaSOM^®^), Pharmaceutical Sciences Graduate Program, Faculty of Pharmacy, Federal University of Rio Grande do Sul, Porto Alegre 90610-000, Brazil; gus.mneves@gmail.com; 2 Faculty of Medicine, Federal University of Rio Grande, Rio Grande 96200-200, Brazil; 3 Department of Pharmacy, Health Sciences Centre, Federal University of Santa Maria, Santa Maria 97105-900, Brazil; graziela.lock@gmail.com; 4 Graduate Program in Integral Health Care (UNICRUZ/URI-Erechim/UNIJUÍ), Integrated Regional University of Alto Uruguai e das Missões, Erechim 99709-910, Brazil; itamar3141@yahoo.com.br; 5 Pharmacokinetics and PK/PD Modeling Laboratory, Faculty of Pharmacy, Federal University of Rio Grande do Sul, Porto Alegre 90610-000, Brazil; dalla.costa@ufrgs.br

**Keywords:** quinolones and fluoroquinolones, drug development and history, pharmacokinetics, bibliometric analysis, molecular docking

## Abstract

Quinolones are among the most successful antibacterial drugs developed in the last century. These drugs act selectively by inhibiting bacterial type II topoisomerases (DNA gyrase and topoisomerase IV), thereby impairing DNA replication and transcription. In the more than sixty years since the first quinolone entered the market, several others have been developed and classified into four generations according to their chemical modifications and effects on the spectrum of antibacterial activity. This review aims to provide an updated overview on this class of antimicrobials for scientists and clinicians doing research in the field of quinolones. We considered perspectives from several areas of medicinal chemistry, including the historical development and chemical evolution of quinolones, as well as their pharmacodynamics and mechanism of action, pharmacokinetics, and toxicity. Additionally, we integrated modern computational and analytical approaches, including bibliometric analysis, molecular docking, and drug-likeness profiling of quinolones to further understand their evolution and development.

## 1. Introduction

The Quinolones are among the most successful and clinically relevant antibacterial drugs developed in the last century. The term quinolone is a generic name given to synthetic antimicrobial drugs that have activity against Gram-negative and Gram-positive microorganisms [1]. These drugs inhibit bacterial type II topoisomerases, DNA Gyrase and Topoisomerase IV (Topo IV), thereby impairing DNA replication and secondarily affecting transcription [1]. Therefore, they are drugs with a selective mechanism of action, acting only against bacteria, since eukaryotes do not express these two enzymes.

**Figure 1:**
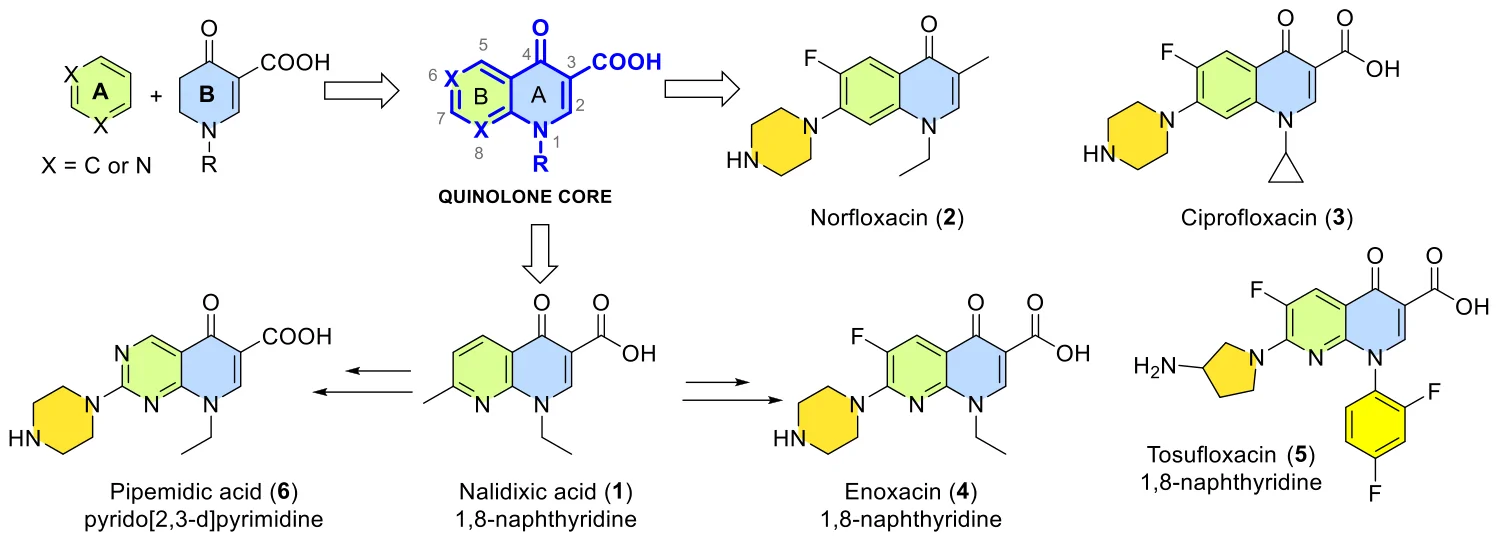
The two patterns of quinolone scaffold: quinolone (**2** and **3**) and 1,8-naphthyridines (**1**, **4** and **5**). Blue bonds indicate the conserved pharmacophoric region of the quinolone core.

The first quinolone entering the market in the 1960s to treat Gram-negative urinary tract infections (*Escherichia coli*) was nalidixic acid 1 (NA), commercially known as Neggram^®^, produced by Sterling Drug Inc (USA) (Figure 1) [2,3]. From then on, several laboratories began to produce other quinolones with superior pharmacodynamic (PD) and pharmacokinetic (PK) properties, as will be discussed in this review. The fact that these compounds are obtained exclusively by chemical synthesis brought hope to medicinal chemists at the time of having new antimicrobials with a pharmacophore other than beta-lactam rings. Furthermore, an additional advantage was the possibility of synthesizing hundreds of derivatives, with modifications in various positions of this bicyclic system, differently than the penicillin group, the most potent antibacterial drugs available in therapy at that time, whose chemical synthesis was much more complex.

The therapeutic utility of quinolones increased dramatically in the early 1980s [4,5] due to the discovery that substitutions of quinolones (or 1,8-naphthyridinones) at C6 and C7 with both fluorine atom and a piperazine, respectively, led to more potent compounds with a broader spectrum of antibacterial activity. These 6,7-substituted quinolones allowed the treatment of many Gram-negative bacteria, including *Pseudomonas aeruginosa*, for instance. Norfloxacin **2** (NOR) was the first fluoroquinolone with these two structural characteristics to enter the North American market in the 1980s [6]. NOR served as a template for the synthesis and market entry of quinolones with a better antibacterial profile, used for treating systemic infections.

Although there are many quinolones in use in human clinical practice, with a wider spectrum of action and better pharmacokinetic profiles, many others have been withdrawn from the market in some countries over time. This article mainly addresses the quinolones that are currently on the market in Brazil, United States of America, Germany, France, United Kingdom and Japan (Table 1) [7,8,9,10,11,12]. This is not an extensive review article, but rather addresses some aspects that we consider important from the point of view of medicinal chemistry, including quinolone’s development, mechanism of action, pharmacodynamics, pharmacokinetics, and toxicity. It includes a brief historical overview of the origin of quinolones and their entry into the market over time. Molecular docking with the selected quinolones facilitates a 3D visualization of the mechanism of action, with the main interactions being presented and discussed in the review, allowing the establishment of structure–activity relationship (SAR). Our goal is to provide an updated overview on this class of antimicrobials for scientists and clinicians doing research in the field of quinolones medicinal chemistry.

### 1.1. Chemistry—General Structure

Quinolone scaffold is a bicyclic system consisting of an aromatic ring B (or heteroaromatic) condensed to a pyridone-4-carboxylic-3 acid (ring A) (Figure 1). Thus, chemically we have two types of quinolones: the quinolones themselves and the 1,8-naphthyridones, which are usually called “quinolones”. Figure 1 shows the two differences between them, exemplifying the structural differences with NA **1**, ciprofloxacin **3** (CIP), enoxacin **4** (ENX) and tosufloxacin **5** (TFLX). Additionally, Dainippon Pharm Co. (Japan) presented a quinolone with an additional nitrogen atom at position 6 of ring B, forming the pyrido [2,3-d]pyrimidine ring and its representative, pipemidic acid (PPA) **6**. In the quinolone scaffold, numerous modifications were made at all chemically available positions, resulting in hundreds of different quinolones. Those modifications will be discussed throughout the review, mainly in Section 4.3.1 (Structure–Activity Relationships) and Section 4.3.2 (Structure–Toxicity Relationships), where we discuss key modifications and their implications for pharmacology, pharmacokinetics, and toxicity. However, those that entered the market were quinolones with modifications only at the four positions of the aromatic ring, as well as at the N1 position. The modifications made resulted in drugs with several improvements, both in pharmacodynamics and pharmacokinetics or even toxicity.

### 1.2. Ionization of Quinolones

**Figure 2:**
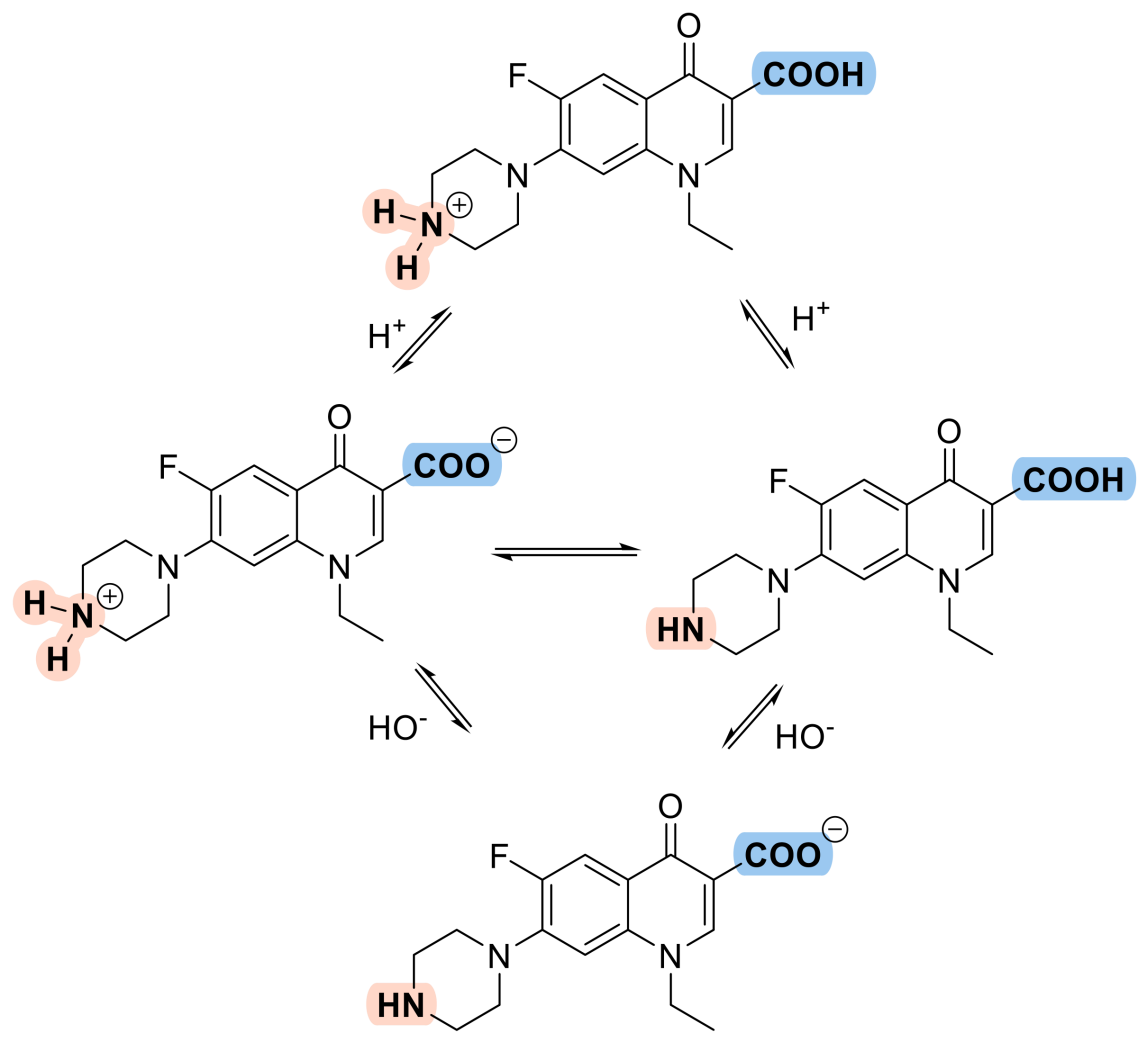
Norfloxacin (NOR) ionization.

Most quinolones are amphoteric compounds. The piperazine ring (or another nitrogen ring) at C7 leads to the formation of a zwitterion in solution or in physiological media, with the existence of two ionization sites. This physicochemical characteristic influences the solubility and absorption of these drugs, impacting the antibacterial activity (greater penetration into the bacterial cell) [1]. Figure 2 exemplifies this characteristic with NOR, where the zwitterionic form predominates at neutral or weakly acidic pH, the protonated form of piperazine at acidic pH, and the anionic form with deprotonation of the carboxylic acid at C3 in basic media [13,14]. It was precisely the placement of piperazine at C7 that caused the improvement in the pharmacokinetic profile, because it increased tissue penetration, and this can be explained by the ionization exemplified here.

### 1.3. Classification

Quinolones are classified into generations according to the chemical modifications performed and their impact on the spectrum of action and clinical use [15,16,17]. Thus, currently there are four generations of quinolones; some entered the market in many countries, although some of them were withdrawn or were never marketed in some countries, due to their detected adverse effects. Additionally, we present below a summary of these drugs and their respective classifications.

**First Generation**: NA was used as the lead compound. These drugs present a narrow spectrum of action and are active mainly against Gram-negative strains, being indicated only for urinary tract infections. Modifications were performed to increase the spectrum of activity.
**Second Generation**: These quinolones have both a fluorine at C6 and a nitrogen ring at C7, providing drugs with a slightly better spectrum of activity. NOR was the most active of all existing quinolones, being the first to enter the market with these characteristics. It then became the template to be domesticated and effectively some modifications in C8 and N1 were performed. These modifications gave rise to the so-called second-generation quinolones (even called fluoroquinolones—FQ), with superior Gram-negative activity and active against some Gram-positive microorganisms as well. One can highlight ofloxacin (OFLX), which emerged in the 1980s, that is a quinolone with an additional 1,3-oxacin ring at C8 and N1, active against Gram-positive microorganisms and marketed as racemate. Bioisosterism was one of the strategies employed and this gave rise to rufloxacin (RFX), a drug with a longer half-life than NOR, but with less bactericidal potency.
**Third Generation**: Resolution of the OFLX led to the production of levofloxacin, which proved to be much more active than the racemic mixture. This led to the synthesis of dozens of tricyclic derivatives. It is observed that in all cases the Fluorine in C6 and nitrogen rings in C7 were maintained. The modifications were made in C5, C8 and N1, except for pazufloxacin (PZFX).
**Fourth Generation**: These drugs have activity against microorganisms Gram-positive and Gram-negative, as well as anaerobes. First, one can observe that the strategy of keeping fluorine in C6 and nitrogen rings in C7 was maintained in almost all of the representatives. Chemically, they present new modifications that affect both the spectrum of activity, the duration of effect and the route of administration. The introduction of chiral centers is observed in both the C7 and N1 amino heterocycles. Many have substituents at C7 and are even nitrogenated (N7), constituting 1,8-naphthyridine derivatives. There are also important modifications at position C8 of ring B, such as the addition of donor or electron-withdrawing groups, or even the replacement of C8 with N8.

## 2. Origin and Development of 1st and 2nd Quinolones Generations:

**Figure 3:**
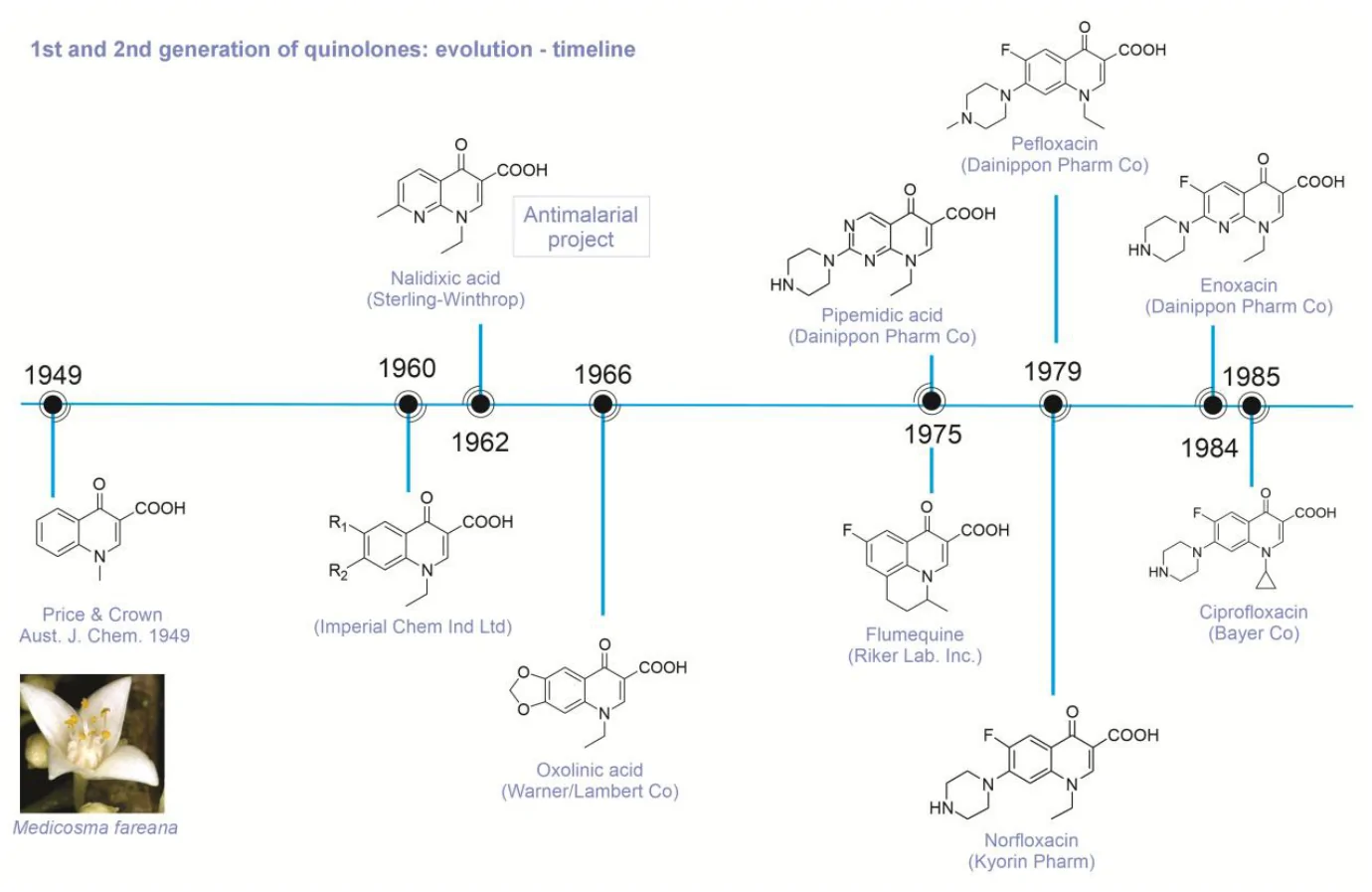
Timeline of the structural evolution of first- and second-generation quinolones, from precursors identified in *Medicosma fareana* and the antimalarial project that led to nalidixic acid, to the development of derivatives such as oxolinic acid, flumequine, pipemidic acid, norfloxacin, pefloxacin, enoxacin, and ciprofloxacin, with the corresponding years and associated researchers/companies indicated. The figure was created using CorelDRAW Graphics Suite 2024.

The literature attributes the discovery of NA to chance by Lesher and colleagues in the 1960s, in which this drug was identified as a by-product in the synthesis of chloroquine [2,3,18,19]. Recently, G. Bisacchi (2015) wrote an excellent manuscript detailing a little more about the discovery of NA, pointing out the existence of other laboratories also interested in quinolones at that time [19]. For greater clarity regarding the origin of quinolones, we conducted a brief literature search, which showed the initial work in medicinal chemistry related to quinolone core and antimicrobial activity. Figure 3 summarizes these findings, which are presented below.

**Figure 4:**
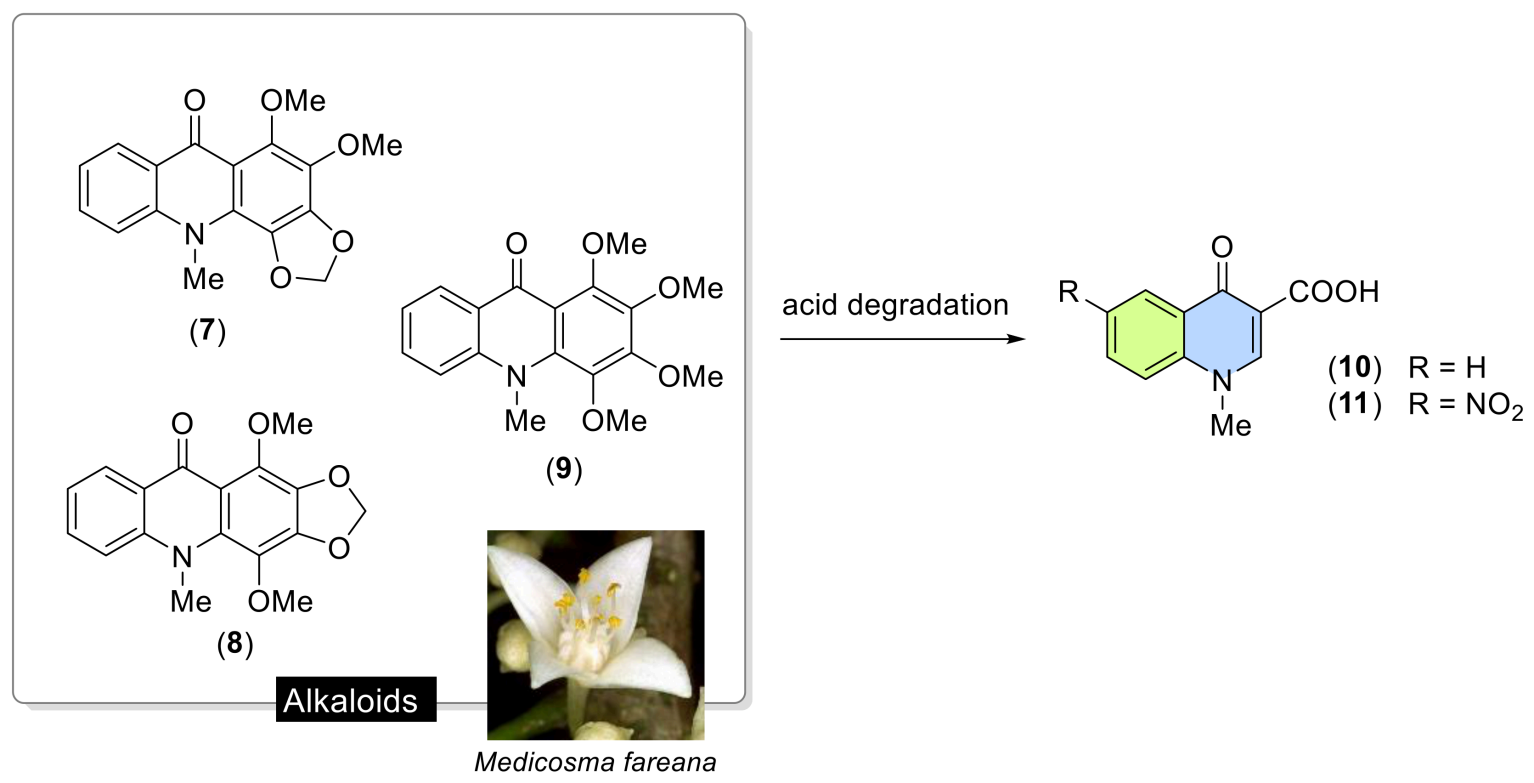
Alkaloids **7**–**9** isolated from *Medicosma fareana* (*Rutaceae*) by Crow and Price [21].

Initially, it is necessary to remember the importance of natural products in medicinal chemistry, with the discovery of new drugs and/or new mechanisms of action over time [20]. The origin of quinolones relates to natural products. The history began in the late 1940s (Figure 3) with the isolation of the alkaloids melicopine **7**, melicopidine **8** and melicopicine **9** from *Melicope fareana* (reclassified as *Medicosma fareana* in 1985 by Thomas Gordon Hartley) by the Australian researchers Crow and Price (Figure 4) [21]. In this work, these alkaloids were isolated and reacted with nitric acid, providing the degradation product **10**. Continuing these investigations, Price performed several experiments with these quinolone alkaloids, obtaining derivatives substituted in both ring A and B, such as nitrated quinolone **11** [22]. None of these compounds had their pharmacological activity investigated; therefore, there were no reports on antimicrobial activity in this report. Over time, new studies were conducted aiming to isolate the other sixty-one quinolone alkaloids from *Waltheria* spp. (Malvaceae), but in none of them was the antimicrobial activity investigated [23].

**Figure 5:**
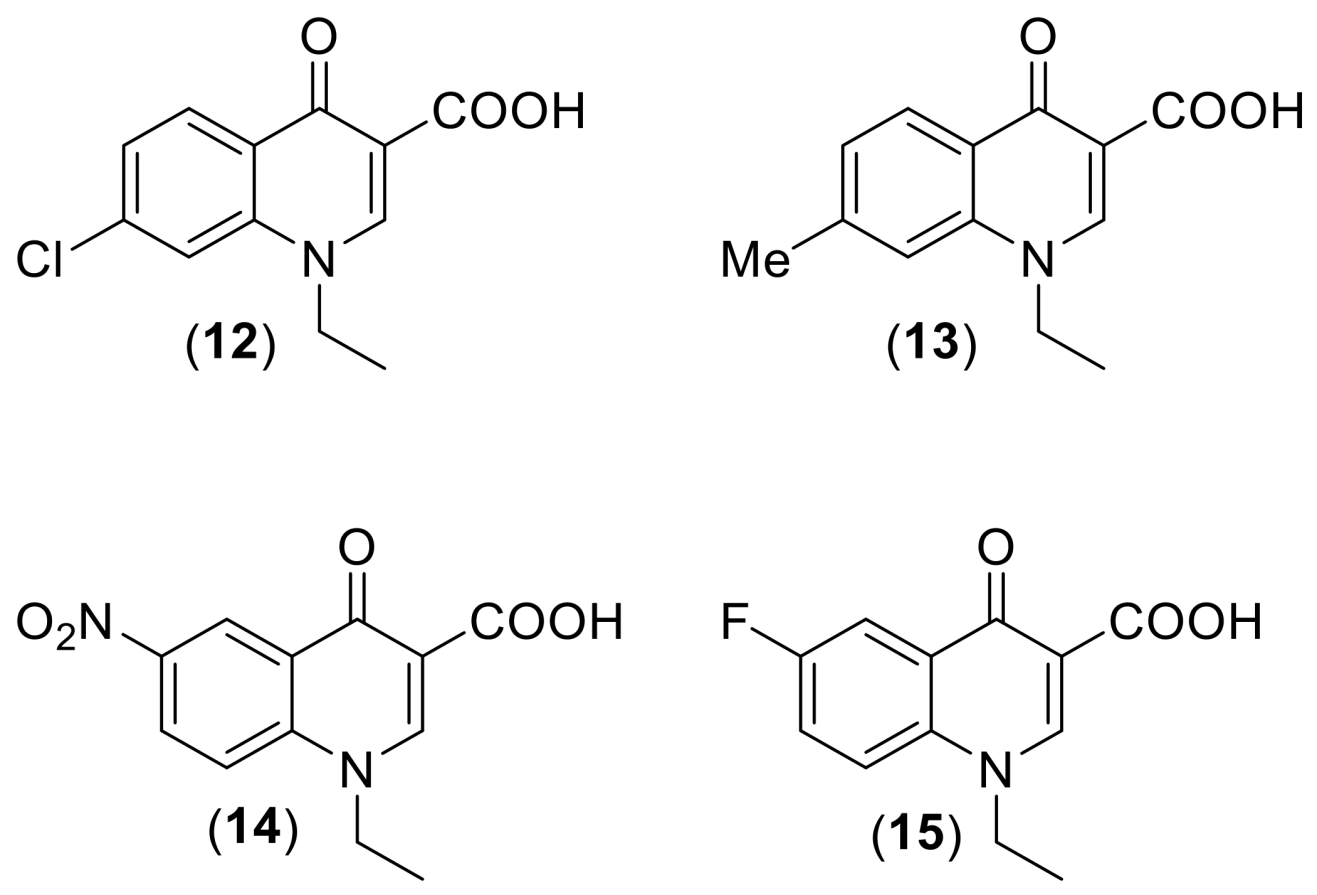
Examples of synthesized quinolones by Hepworth and co-workers in 1960 [25].

In 1960, W. Hepworth and co-workers from Imperial Chemical Industries Ltd. (Slough, UK) patented the synthesis and the antibacterial activity of quinolones with a chemical structure very comparable to compounds **10** and **11**, obtained years earlier [24,25,26]. They made several modifications to the aromatic ring B, such as 7-Cl, 7-Me, and 6-NO_2_ (Figure 5). In the case of N-1, they replaced the methyl by an ethyl group. Interestingly they kept the 4-oxo group and 3-COOH in some of the synthesized compounds. This work was the first report on the antibacterial activity of quinolones. Nevertheless, the antimicrobial activities were not strong enough to get any compound into the market, and the project was abandoned.

**Figure 6:**
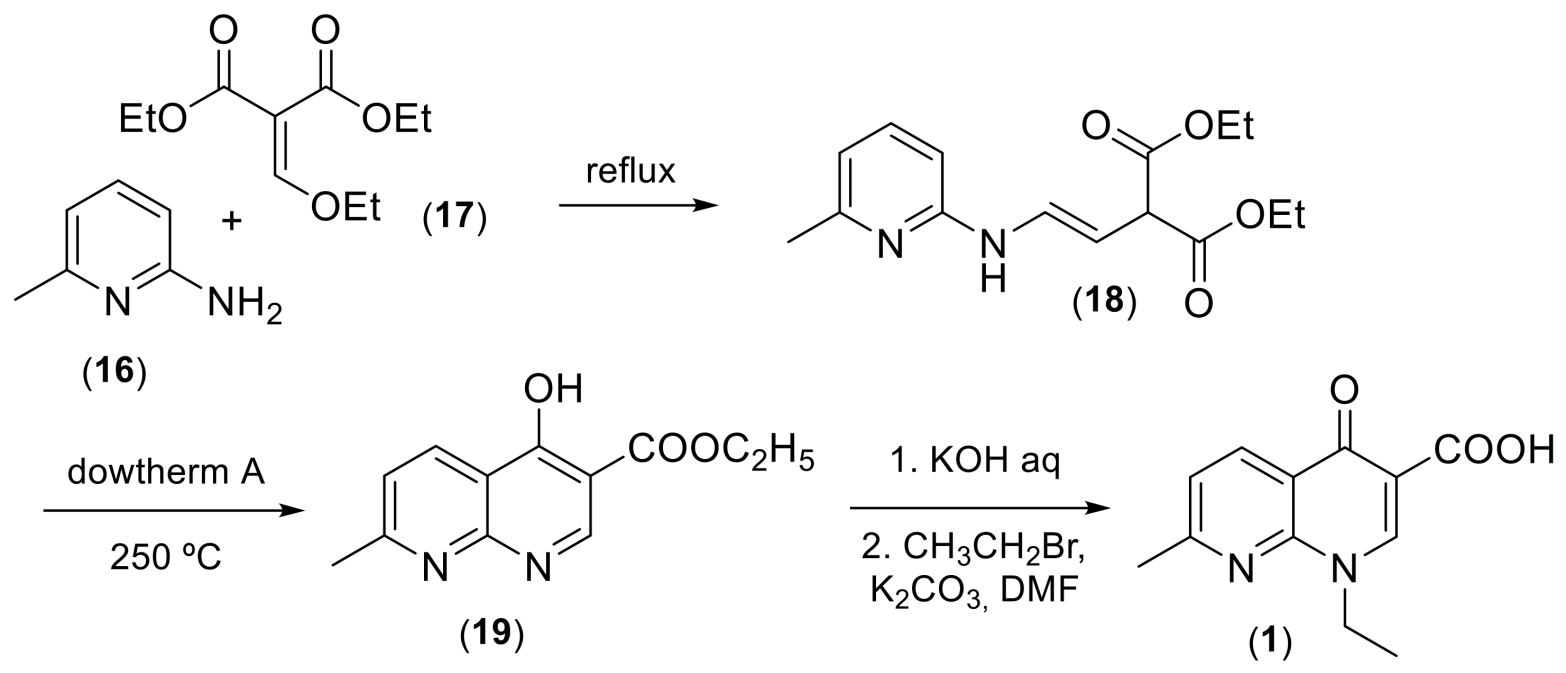
The scheme of nalidixic acid **1** synthesis by Lesher et al., published in 1962 [2,3].

Investigations into this class of heterocycles and their use in therapeutics as antibacterials were intensified in the following years, which led to the emergence of the first quinolone with antibacterial effect against Gram-negative microorganisms to enter the market [6]. In fact, two years later (1962), Lesher and co-workers (Sterling Drug Inc, USA), in an antimalarial synthesis project, published the synthesis of NA 1 (code: WIN 18,320) and its antibacterial properties *in vitro* and *in vivo* [2,3,27]. The synthetic route used by the authors is summarized in Figure 6, which started with 6-methyl-2-aminopyridine 16 in reaction with diethyl ethoxymethylenemalonate **17** at high temperatures, followed by hydrolysis in alkaline medium and alkylation of N-1 [2,3]. This was the first 1,8-naphthyridine synthesized and categorized as quinolone at that time, beginning a new era in the fight against bacterial infections.

**Figure 7:**
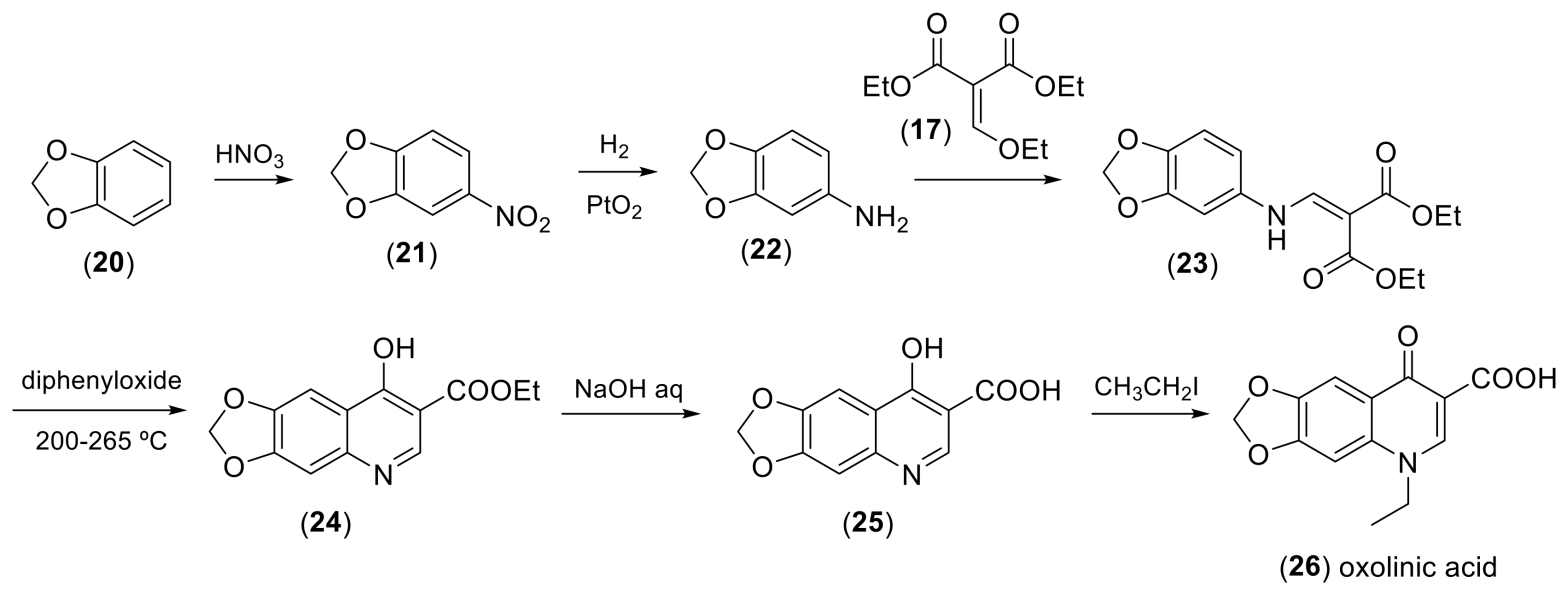
The scheme of oxolinic acid 26 synthesis by Kaminsky et al. [28].

Investigations conducted by Kaminsky and co-workers (Warner-Lambert Co, Morris Plains, NJ, USA) led to the synthesis and antimicrobial evaluation of tricyclic quinolones, such as oxolinic acid **26** (Figure 7) [28,29]. This time, the line of research took another direction and did not adopt the 1,8-naphthyridine system of NA. The structure of this drug seems to be based on previously synthesized compounds **10–11**, whose substituents on the aromatic ring B were replaced by the methylenedioxy fragment. Oxolinic acid was active against Gram-negative bacteria, mainly *Escherichia spp.* and *Proteus spp.*, and was active against NA-resistant strains.

**Figure 8:**
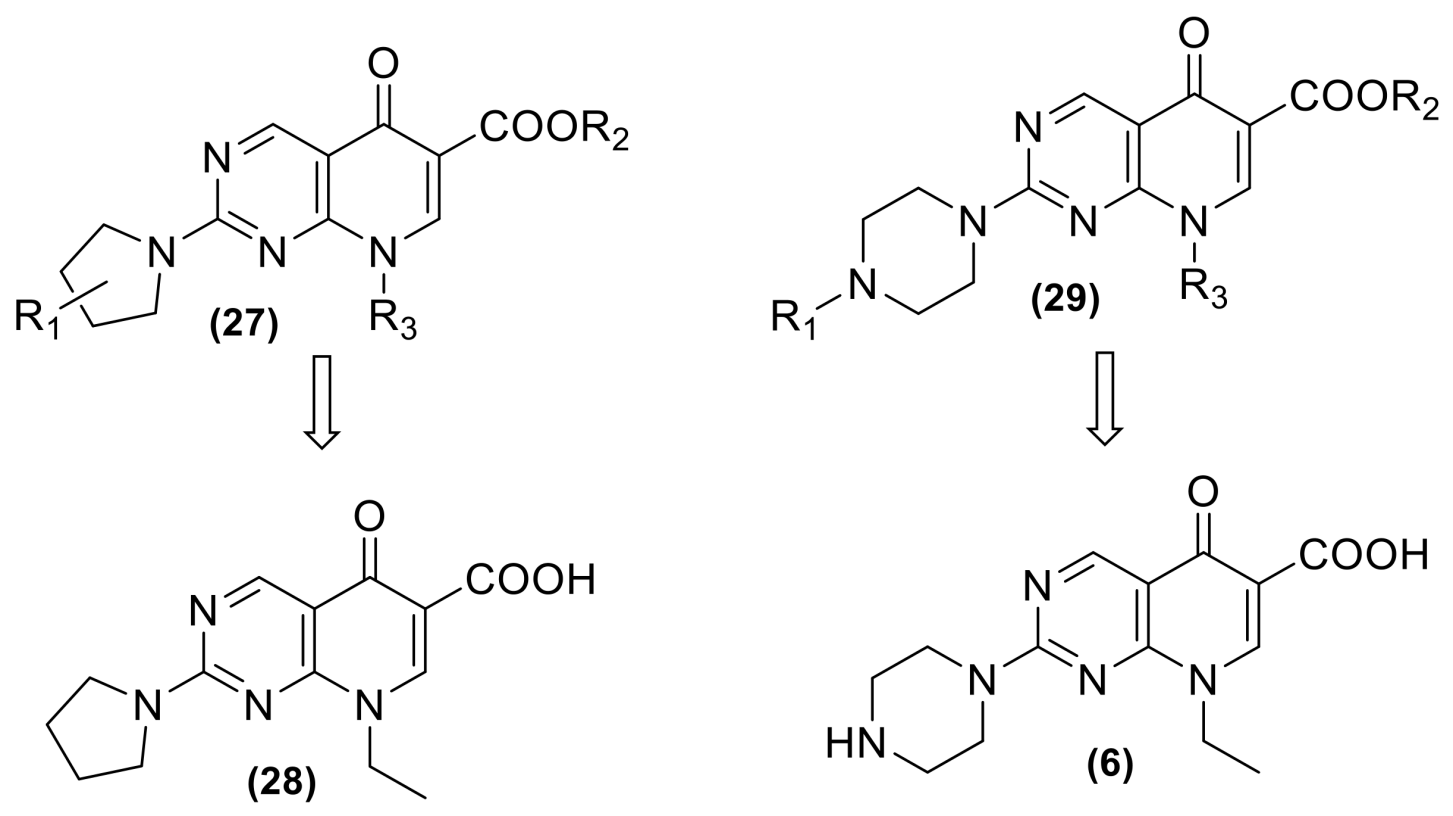
Piromidic acid **28** and PPA **6** origin by Matsumoto in the 1970s [30].

Since the emergence of NA 1, the potential of 1,6 and 1,8 pyrimidines has been investigated and Japanese researchers have explored a new bicyclic system with interesting potential as antimicrobials, pyrido [2,3-d]pyrimidines compounds **27–29** and **6** (Figure 8). Firstly, J.I. Matsumoto and co-workers in 1973 (Dainippon Pharm. Co. Ltd., Osaka, Japan) filed a patent for the synthesis and antimicrobial activity of several 5,8-naphthyridines, 5-oxo, 6-carboxylic with a pyrrolidine ring at 7-position [30]. Among these derivatives, they had piromidic acid **28**, which showed *in vitro* and *in vivo* activity superior to the NA **1** that was on the market. Likewise, it presented excellent intestinal absorption and low toxicity, profiles superior to NA **1** [31]. Continuing these studies, these researchers made an important discovery which was the piperazine linked at 7-position in the pyrido [2,3-d]pyrimidines in place of pyrrolidine. In fact, these researchers replaced pyrrolidine at C7 by piperazine, which led to the production of several compounds with improved antibacterial action, including PPA **6** (Pipram^®^) [32,33,34]. This quinolone was a real breakthrough in the treatment of urinary tract infections, showing superior antibacterial activity to NA **1**, as it was active against both Gram-negative microorganisms and strains resistant to it, mainly *P. aeruginosa* and *Mycobacterium tuberculosis*. PPA **6** showed progress in the treatment of urinary infections, with slower metabolism than oxolinic acid **26**, high urinary concentrations, but still without the ability to treat systemic infections.

In the 1970s, the search for quinolones with a broad spectrum of activity allowing the treatment of systemic infections, with better pharmacokinetic and toxicological profiles, was underway. In contrast with the research with the pyrido [2,3-d]pyrimidines systems, researchers at Kyorin Pharm. Co. (Japan) went in another direction [35,36,37]. They synthesized a set of several derivatives with the quinolone ring instead of pyrido [2,3-d]pyrimidine nucleus. In this project, they added a fluorine atom at C6 and kept the piperazine at C7, which revealed an important discovery, since the addition of this halogen led to a quinolone with superior antibacterial activity to all previous ones, the NOR **2** (code AM-715). Later, in 1980, Kyorin licensed this drug to Merck. Norfloxacin was active against both Gram-positive and Gram-negative bacteria, including *Pseudomonas* spp.

**Figure 9:**
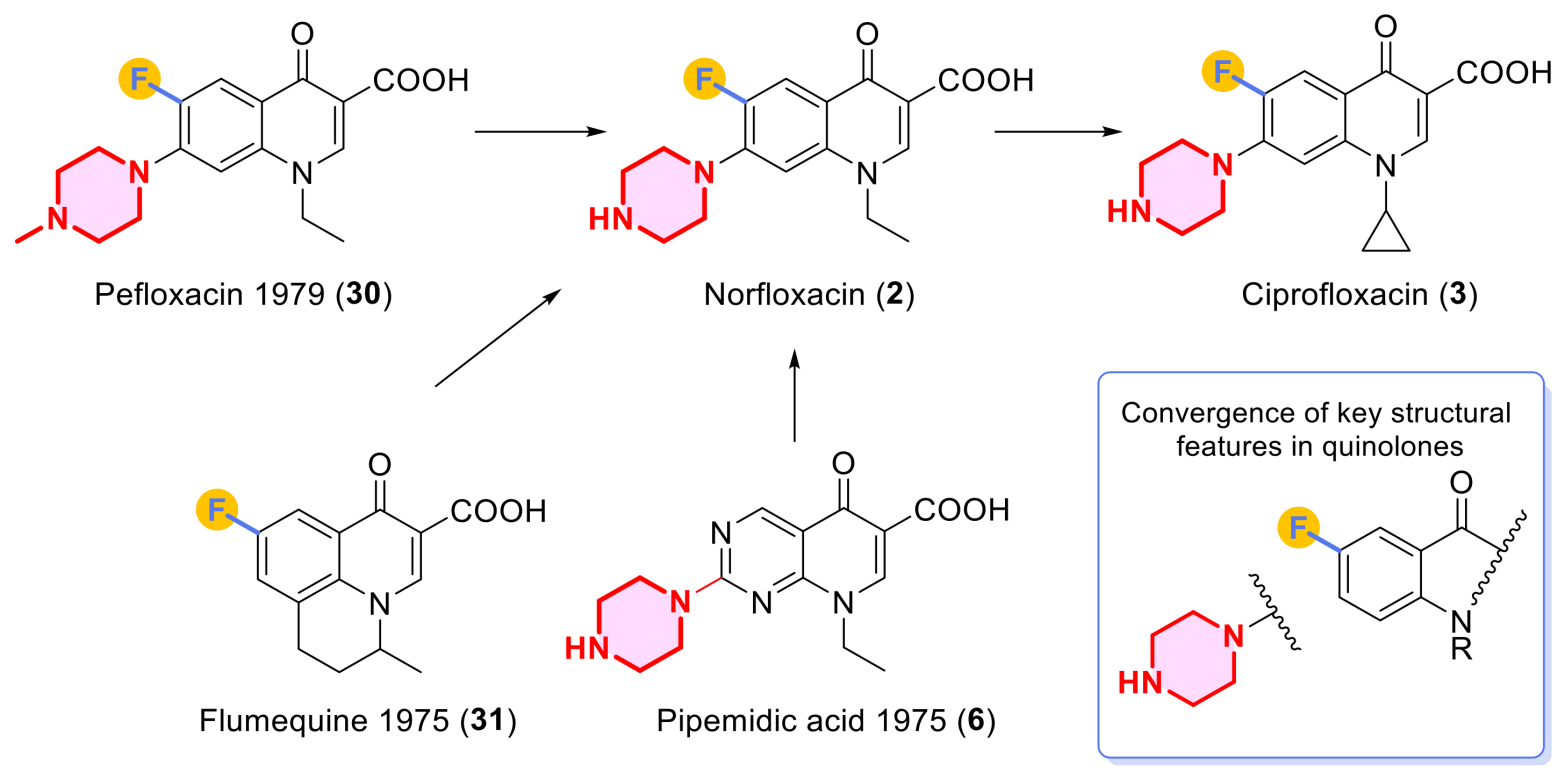
The origin of fluoroquinolones (FQ): a combination of structural features from FLU and PPA.

From this work, second-generation quinolones emerged, giving rise to the term FQ. Figure 9 illustrates the origin of the FQs: flumequine 31 (FLU) has 6-F and PPA 6 has piperazine-7; the union of these two fragments gave rise to, in addition to NOR 2, Pefloxacin (PFL) 30 and Ciprofloxacin (CIP) **3** [38,39,40].

**Figure 10:**
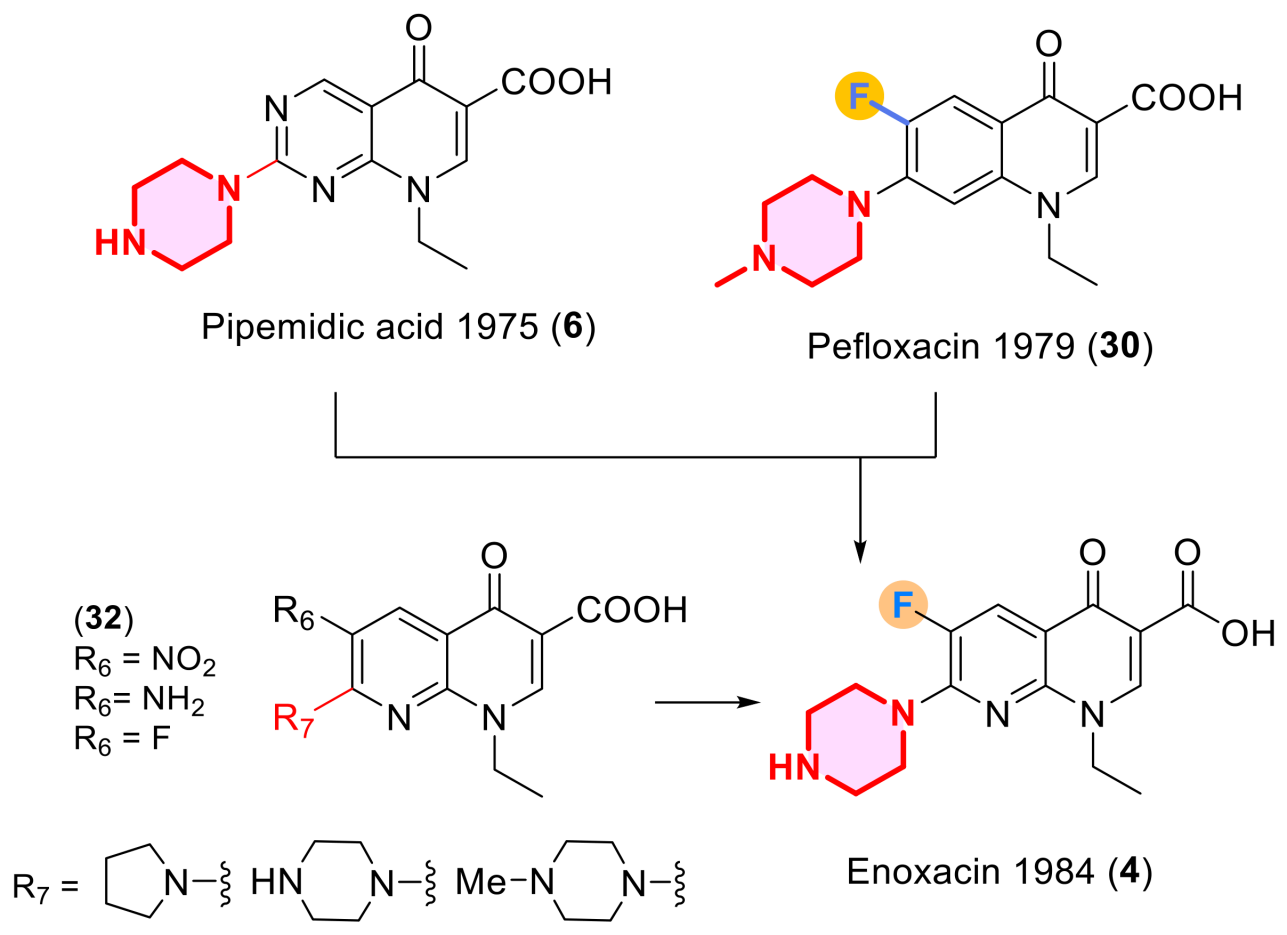
Enoxacin development by Matsumoto et al. [41].

In 1984, Matsumoto and researchers at Dainippon Pharm. Co. developed Enoxacin **4** (ENX), whose pharmacophore is the 1,8-naphthyridine ring found in NA **1** [41]. The project was based on PPA 6 and PFL **30**, and the fragments fluorine and piperazine (Figure 10). These fragments were compared with others, such as NO_2_ and NH_2_ at position 6; in addition to N-methylpiperazine or pyrrolidine at C7 **32**, but no modification produced compounds with a better activity.

In the early 80s, researchers at Daiichi Pharm (Japan) synthesized tricyclic quinolones with a novel scaffold: pyridobenzoxazine, leading to Ofloxacin (OFLX) **35** [42,43,44,45]. This oxazinoquinolone has shown significantly superior activity against various Gram-positive and Gram-negative bacteria, as well as some anaerobic bacteria, in addition to a longer half-life. Oral and intravenous administration was possible, and it is indicated for systemic infections. OFLX was first patented in Japan as Tarivid^®^ (Daiichi Lab) and licensed in subsequent years to Hoechst (European market) and Johnson & Johnson (American market).

**Figure 11:**
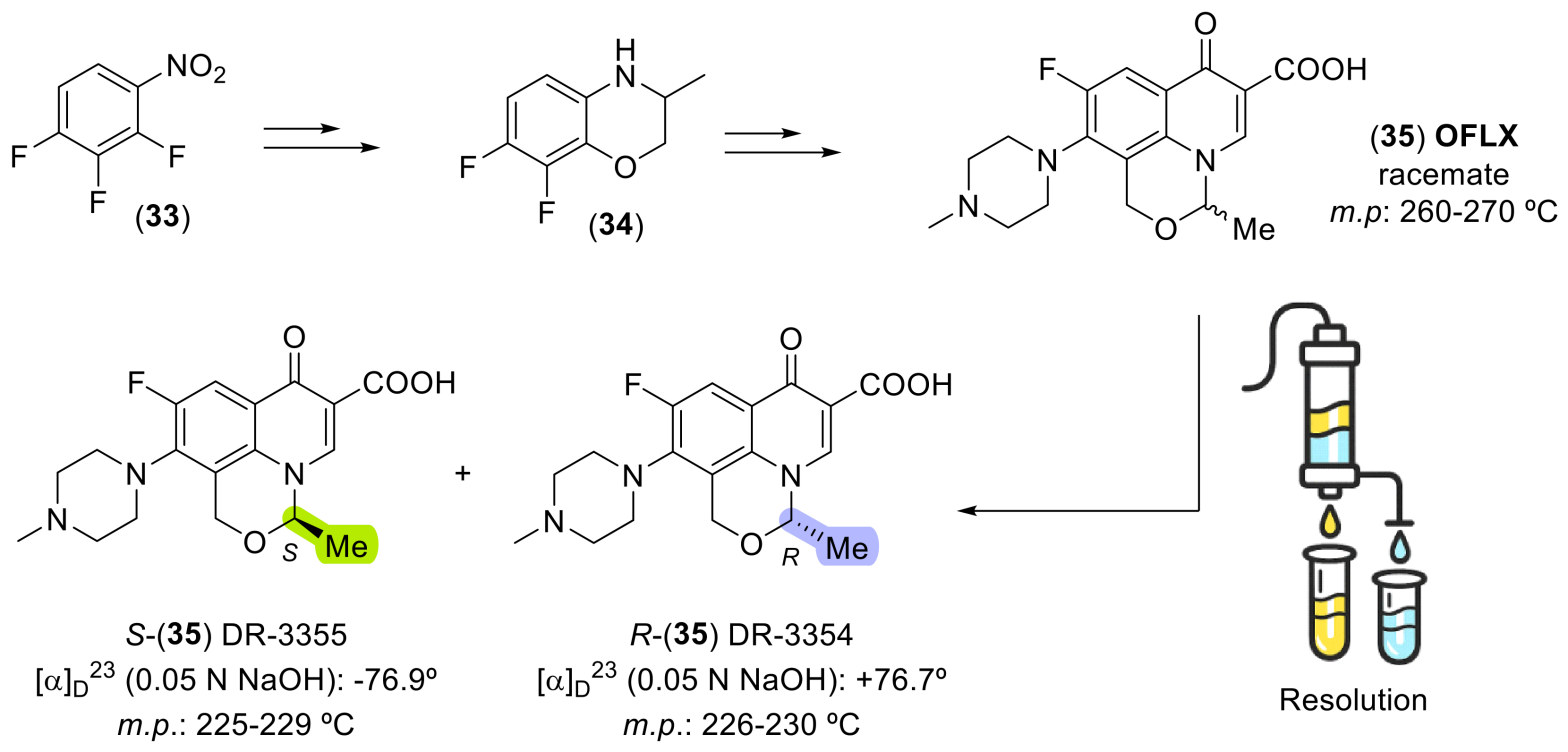
Scheme of OFLX synthesis and resolution into its enantiomers.

However, this drug has an asymmetric carbon (C3), originated from its synthesis, and is marketed as a racemic mixture. It is a synthesis based on the Gould and Jacob method, which begins with 2,3,4-trifluoronitrobenzene, building the oxacin ring first, then the 4-oxo-3-carboxypyrimidone ring, and introducing N- methylpiperazine to form OFLX (Figure 11).

The researchers rapidly realized the need to obtain the two enantiomers separately in order to test them and select the most active one. After much effort, the *R* and *S* isomers were separated from OFLX derivative racemate by HPLC with a chiral stationary phase [45]. However, the first resolution yielded only 2.5 mg of each enantiomer, which was too small for biological and physicochemical tests. Several separations and one month of work were necessary to obtain the required quantities of each isomer (250 mg) to be investigated. Figure 11 shows the measured melting points for the racemate OFLX and the two enantiomers, as well as the refractive indices of both isomers, confirming the separation of these two compounds.

For comparison, the enantiomers *R* and *S* were tested separately along with the racemate (OFLX). The results in Table 2 show that the *S* enantiomer (later called Levofloxacin—LEV) exhibited twice the activity of the racemate in six bacterial strains tested and the same magnitude in four others. The variation in activity between the *S* and *R* depends on the strain and ranges from 7.8 times in *E. coli* and *Proteus vulgaris* to 125 times for *Staphylococcus aureus* and *Staphylococcus* epidermidis, with the *R* distomer showing no activity against *Streptococcus faecalis* and *Streptococcus pyogenes*. It was also observed that the *S* enantiomer is more soluble than the racemate, which facilitates using a lower dose. However, the most important and anticipated finding was that the *S* enantiomer always showed greater inhibition than its optical antipode *R*, regardless of the strain tested, confirming the different behavior of the two enantiomers (*R* and *S*) in a chiral environment, such as that of living organisms. These results encouraged Daiichi researchers, who then patented LEV in 1987 for topical, oral, and intravenous use. Currently, there are different methods in the literature for both OFLX resolution [46] and asymmetric LEV synthesis [47].

## 3. Analysis of the Scientific Production

As observed, quinolones have been the subject of research and development since the mid-20th century, with hundreds of derivatives synthesized over time by various research groups around the world. The pursuit has always been for an antibacterial agent with a broad spectrum of action against Gram-positive and Gram-negative bacteria, anaerobes, and resistant strains such as MRSA. Similarly, efforts have been made to improve pharmacokinetics to allow for easier dosing regimens such as single daily doses for the treatment of systemic infections. Therefore, many quinolones have entered the market, while many others have been withdrawn due to problems, primarily toxicity issues or insufficient PK profiles. In a survey conducted by Newman and Craig [20] on antibacterial drugs approved by the FDA from January 1981 to September 2019, it was found that out of a total of 162 approved antibacterial, 36 were fully synthetic drugs, and of these, 30 belong to the quinolone class, representing 18.5% of the antibacterial approved in this 40-year period, which is a significant number.

Aiming for a more detailed assessment, a bibliometric analysis in the Web of Science database was conducted between 1945 and 2025. The search performed identified 21,536 publications, including original and review articles, focused on specific antibacterial quinolone drugs. The complete search strategy is available in the electronic Supplementary Materials. To investigate the evolution of this drug class and identify key milestones in its scientific development, the records were exported as *BibTeX* files and the temporal dynamics of title words were analyzed using the *Bibliometrix* package in the *R* environment [48].

**Figure 12:**
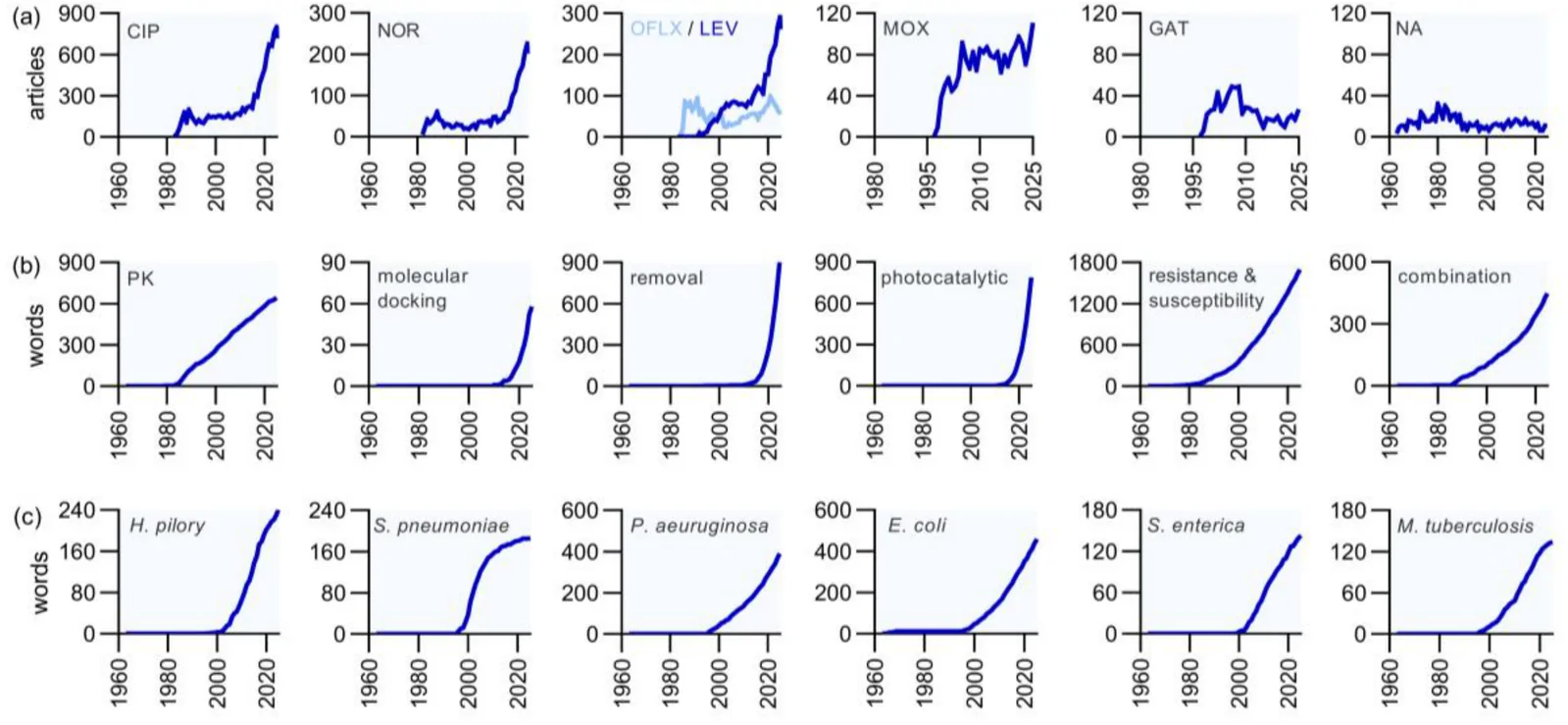
Word dynamics trends in quinolone-related research from 1945 to 2025: in (**a**) annual publication counts for selected quinolones: ciprofloxacin (CIP), norfloxacin (NOR), ofloxacin (OFLX), levofloxacin (LEV), moxifloxacin (MXF), gatifloxacin (GAT), and nalidixic acid (NA); in (**b**) temporal dynamics of frequent title terms associated with major quinolone research topics, including pharmacokinetics, molecular docking, environmental removal, photocatalytic degradation, resistance/susceptibility, and combination approaches; in (**c**) temporal trends in quinolone-related publications mentioning key bacterial pathogens, including *H. pylori, S. pneumoniae, P. aeruginosa, E. coli, S. enterica*, and *M. tuberculosis*. Data were collected from the Web of Science database in May 2026. The figure was created using GraphPad Prism 10.

Regarding the selected quinolones, the publication dynamics indicate distinct historical patterns across the class. Although CIP, NOR, and OFLX were developed before the 1990s, a more pronounced increase in scientific attention was observed mainly from the 2010s onward (Figure 12a). In the combined OFLX/LEV panel, the scientific production related to LEV increased after that of OFLX, consistent with the fact that LEV is the (S)-enantiomer of OFLX, which was originally developed and investigated as a racemic mixture. In contrast, publications related to MXF and GAT began to increase around the mid-1990s, reflecting the later introduction and evaluation of these newer fluoroquinolones (Figure 12a). For LEV and MXF, the sustained increase after 2000 may also reflect their growing importance in drug-resistant tuberculosis research and treatment optimization, as later-generation fluoroquinolones became key components of multidrug resistance regimens in tuberculosis [49,50]. NA, the first representative of the class, showed an earlier publication peak around the 1980s, followed by a decline and persistently lower scientific output compared with the other quinolones analyzed. This trend may reflect historical factors, including changes in the pace of antimicrobial discovery and the increasing global concern regarding bacterial resistance [51,52]. The recent growth in publications on several quinolones may also have been influenced, at least in part, by patent expiration, which may have facilitated broader production, accessibility, and academic investigation.

The temporal dynamics of selected title terms further reveal the diversification of quinolone research over time (Figure 12b). Some areas appear to have been strongly influenced by technological advances. For example, publications involving pharmacokinetics increased markedly from the 1980s onward, whereas terms related to molecular docking became more prominent mainly after the 2010s. The expansion of *in silico* studies is likely associated with the increasing availability of structural information on bacterial targets, including crystallographic studies of DNA gyrase and Topo IV, which provided templates for docking and structure-based investigations [53,54].

The keyword dynamics also highlight the growing environmental relevance of quinolones. The increasing frequency of terms such as “removal” and “photocatalytic” indicates rising concern about the persistence of these compounds in aquatic and environmental compartments, as well as growing interest in advanced remediation strategies for eliminating quinolone residues from wastewater and related matrices (Figure 12b). This interpretation is supported by studies showing that fluoroquinolones can be strongly transferred from wastewater to sewage sludge and persist for months in sludge-treated soils [55], together with the marked expansion of research on antibiotic occurrence, fate, and effects in aquatic systems during the 2000s [56]. The subsequent rise of “photocatalytic” is also consistent with the development of heterogeneous photocatalysis as an approach for degrading ciprofloxacin in contaminated waters, an area that expanded substantially during the following decade [57]. In parallel, antimicrobial resistance remains a central and expanding topic in quinolone research, as shown by the increasing occurrence of terms related to “resistance and susceptibility” and “combination” (Figure 12b). These trends suggest sustained interest in resistance mechanisms, susceptibility profiling, and combination-based approaches aimed at preserving or restoring quinolone efficacy against resistant bacterial strains.

A marked increase in the occurrence of specific bacterial names in article titles was also observed from the late 1990s and early 2000s onward, including *Helicobacter pylori, Streptococcus pneumoniae, Pseudomonas aeruginosa, Escherichia coli, Salmonella enterica*, and *Mycobacterium tuberculosis* (Figure 12c). This pattern likely reflects the clinical relevance of these pathogens, the expansion of quinolone indications, and the growing need to evaluate antimicrobial activity against organisms associated with resistance or difficult-to-treat infections. Advances in molecular diagnostics and the establishment of antimicrobial resistance surveillance programs may have further stimulated pathogen-focused studies. The increase in M. tuberculosis-related publications after the late 1990s is consistent with the expanding clinical role of fluoroquinolones in drug-resistant tuberculosis. By 2003, fluoroquinolones were already used primarily when resistance or intolerance to first-line therapy occurred [58]. During the following decade, fluoroquinolones were described as cornerstone components of MDR-TB regimens [49], and an individual-patient meta-analysis of 9,153 cases associated later-generation quinolones with improved treatment success and survival [50]. Furthermore, WHO guidelines recommended the inclusion of fluoroquinolones, preferably later-generation agents, in MDR-TB treatment regimens [59].

## 4. Pharmacodynamics

### 4.1. Mechanism of Action

**Figure 13:**
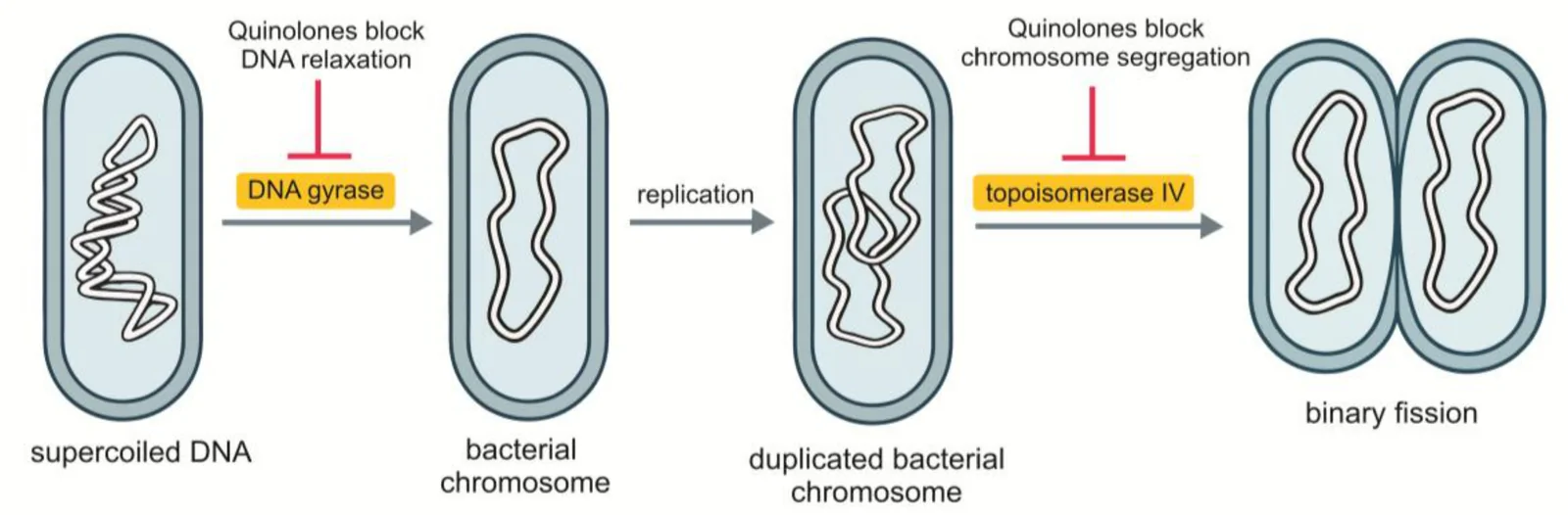
Diagram of the mechanism of action of quinolones. The figure was created using CorelDRAW Graphics Suite 2024.

Didactically, it can be stated that quinolones bind at the interface between DNA-enzyme and act by inhibiting two bacterial type II topoisomerases involved in DNA replication: DNA gyrase and Topo IV. In general, for bacterial cell replication, the supercoiled DNA must be relaxed (Figure 13) [60] DNA Gyrase is responsible for relaxing the DNA, while Topo IV, in turn, catalysis the separation of chromosomes, thus promoting bacterial cell division. Therefore, by inhibiting these enzymes, the bacterial cell division process is stopped, leading to bacterial death. This is the basic strategy of quinolones. Specifically, quinolones stabilize the transient cleavage complex formed between bacterial DNA and type II topoisomerases, thereby preventing the religation of the cleaved DNA strands. This “trapping” of the cleavage complex leads to the accumulation of double-strand breaks, ultimately blocking replication and transcription and driving bacterial cell death. For more detailed information regarding topoisomerases, readers are encouraged to consult the following references: [61,62].

**Figure 14:**
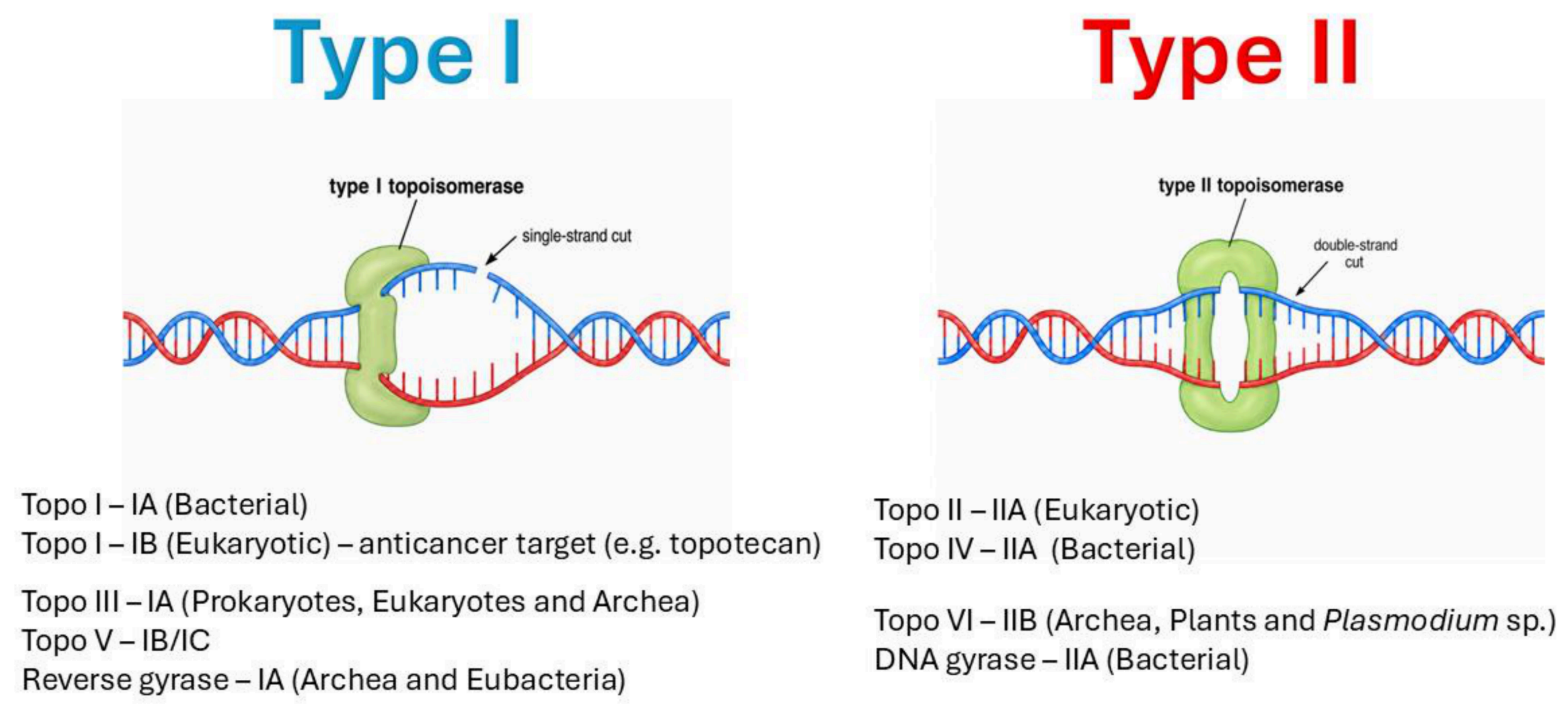
Topoisomerase classification according to the type: Type I catalyze single-strand breaks, whereas Type II catalyze double-strand breaks. The figure was created using GIMP (Version 3.2).

Topoisomerases are classified into Type I, which catalyze single-strand breaks and Type II, which generate double-strand breaks (Figure 14). While this review focuses mainly on bacterial topoisomerases (Gyrase and Topo IV), it is important to highlight some pharmacologically and clinically relevant human counterparts for comparison. Among eukaryotic type I topoisomerases, topoisomerase IB relaxes both positively and negatively supercoiled DNA and is structurally distinct from bacterial type IA enzymes. Topoisomerase IB is a clinically relevant target for quinoline alkaloids used as antitumor agents, such as camptothecin, irinotecan, and topotecan [63]. As outlined above, the topoisomerases are a key family of enzymes responsible for modulating DNA packing dynamics. These enzymes alter DNA topology by introducing temporary strand breaks, allowing controlled passage of DNA segments, and then resealing it. The main alterations of DNA topology include relaxation, supercoiling, decatenation, catenation, knotting and unknotting.

Regarding the type II topoisomerases, they are responsible for relaxing both negatively and positively supercoiled DNA, using ATP as an energy source and Mg^2+^ as cofactor. The Topo II is also clinically important target for chemotherapeutics (non-covalent poisons) including the podophyllotoxin derivatives etoposide and teniposide, and the anthracycline chemotherapeutic doxorubicin [60,64]. Despite homology with bacterial type II enzymes (DNA gyrase and Topo IV), eukaryotic Topo II enzymes differ particularly in their C-terminal domains, which appears to be mechanistically important for the bacteria. While eukaryotic type II topoisomerases play essential roles in chromosome dynamics, their bacterial counterparts possess unique functional specializations that are critical for bacterial viability and represent key targets for antibacterial drug development [65]. Both bacterial enzymes will be further described in the following sections.

The bacterial lethality of quinolones is associated with two mechanisms depending on the exposure concentration: (a) slow death and (b) rapid death. The slow death occurs when the concentrations are at least twice the minimum inhibition concentration (MIC) leading to bacterial death after an overnight exposure to quinolones. The potential aspects related to this process may occur when the cleavage complex is not completely solved (i.e., the quinolone is still stabilizing the complex, and the topoisomerase is not removed) leading to DNA replication and transcription stalling and consequently cell death [65]. In this scenario, there is an accumulation of positive supercoils ahead of replication fork by DNA Gyrase blockage as well as the non-separation of the daughter chromosomes caused by Topo IV inhibition giving rise to lethal cell-division [66]. On the other hand, the rapid death is triggered when quinolone-topoisomerase complex is either removed, or the topoisomerase subunits are dissociated, while the DNA is left broken resulting in chromosome fragmentation [65]. Moreover, the accumulation of DNA strand breaks may exacerbate the reactive species of oxygen (ROS) levels causing even more DNA damage [65], which leads the bacterial cell to activate DNA repairing mechanisms or to activate apoptosis signaling [66].

#### Structural Overview of DNA Gyrase and Topoisomerase IV

Structurally, DNA Gyrase is a heterotetramer (A_2_B_2_) composed of two GyrA and two GyrB subunits. GyrA contains an N-terminal DNA breakage domain (tower/shoulder, WHD with the catalytic tyrosine, and coiled-coil) and a C-terminal domain forming the C-gate. GyrB contains an N-terminal ATP-binding domain, a transducer domain, and a C-terminal domain with the Topoisomerase-Primase (TOPRIM) domain and tail region [62]. The TOPRIM domain coordinates Mg^2+^ via conserved aspartates, contains a catalytic glutamate, and interacts with subunit A and DNA [62,67,68].

Topo IV is a bacterial type IIA topoisomerase that works in concert with DNA gyrase, although some organisms express only DNA gyrase (e.g., M. tuberculosis, H. pylori and Treponema pallidum) [66]. It relaxes both positive and negative supercoils but cannot introduce negative supercoils. Its main function is decatenation and untangling of interlinked DNA, essential for chromosome segregation after replication. Structurally, Topo IV is a heterotetramer, homologous to DNA gyrase, with differences at the C-terminal domain of the A subunit (ParC/GrlA). Nomenclature varies: in Gram-negative bacteria it consists of two ParC and two ParE subunits, while in Gram-positive bacteria it consists of two GrlA and two GrlB subunits.

### 4.2. Molecular Docking

In this review, we intended to verify how quinolones interact with the complex, as well as to overview the pharmacophoric features relevant for the activity. Therefore, two molecular modeling approaches were applied to 20 quinolones to identify the functional groups most relevant for activity. For the sake of clarity, the detailed methodology is provided in the Supplementary Materials. The assay involved molecular docking simulations with DNA gyrase and Topo IV to evaluate their interactions with the enzymes’ binding sites and with DNA—the respective docking scores and interaction profile were reported in Supplementary Table S1. The docking procedures were validated through redocking, on which all the calculated poses from the cocrystallized ligands (moxifloxacin for *E. coli* DNA gyrase and delafloxacin for *S. pneumoniae* Topoisomerase IV) showed RMSD values within the 2 Å cutoffs (Supplementary Table S1).

**Figure 15:**
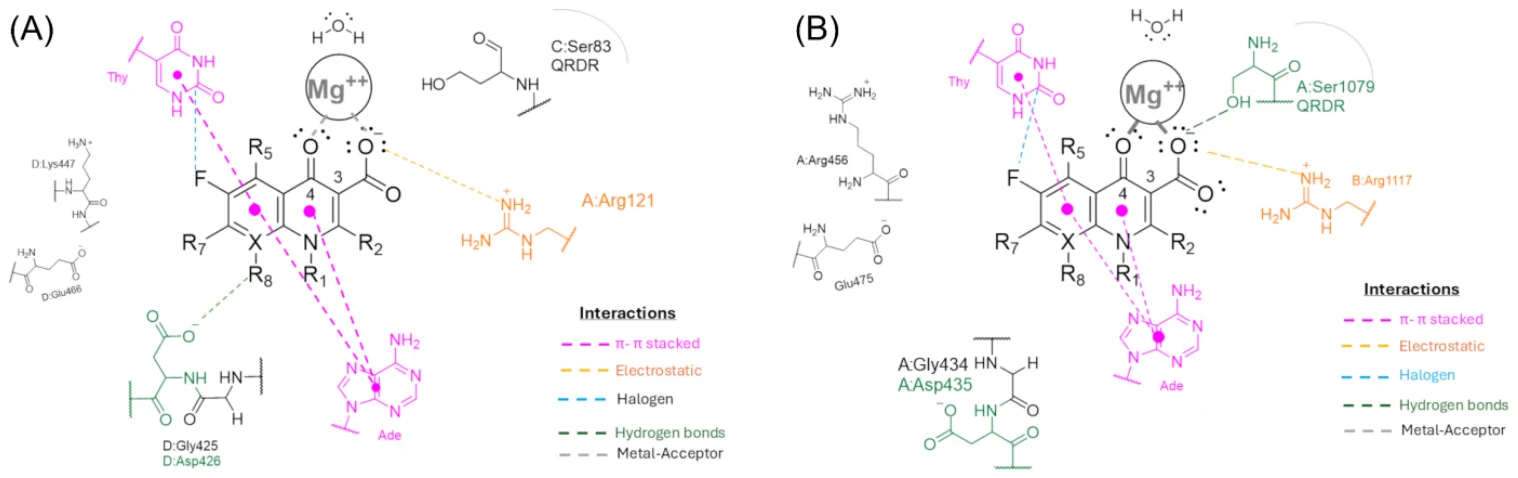
Schematic representation of the protein–ligand interaction of the 20 quinolones docked on: (**A**) *E. coli* DNA gyrase (PDBID: 9GGQ) and (**B**) *S. pneumoniae* topoisomerase IV (PDBID: 8QMB). The main interactions were represented in dashed lines and colored according to their chemical nature: π-π stacked (pink), electrostatic (orange), halogen bonds (cyan), hydrogen bonds (green) and metal–acceptor interactions (grey). The 2D molecular interaction diagrams were generated using BIOVIA Discovery Studio Visualizer.

Regarding the docking evaluation on DNA gyrase (*E. coli*, PDBID: 9GGQ) (Figure 15A, Supplementary Figure S1 and Table S1), the best scored poses from each quinolone showed scoring values from 81.8371 (premafloxacin) to 118.133 (trovafloxacin). The cocrystallized ligand (moxifloxacin) displayed a ChemPLP score of 102,026. The binding interaction analysis revealed that all the quinolones bind in a similar manner, with the following main interactions:

(1) The quinolone scaffold is sandwiched between two deoxyadenosines (Ade1 and Ade18) and performing π-π interactions with two deoxythymidines (Thy4 and Thy5)—in pink in the figure.
(2) The carboxylate and the carbonyl groups at positions 3 and 4 of the quinolone ring may perform electrostatic interactions with Mg^2+^—grey dashed line.
(3) The carboxylate group at position 3 may interact with Arg121 from GyrA subunit through hydrogen bonds and electrostatic interactions—orange dashed line.
(4) The fluorine group at position 6 may interact with the carbonyl group of Thy 4 and 5 through halogen bonds—cyan dashed line.

Further interactions observed in were found with GyrB subunit involving Gly425, Asp426, Lys447 and Glu466 regarding the substituents at positions 7 and 8, which may perform van der Waals and hydrogen bonds—green dashed line. Moreover, although Ser83 does not interact directly with the quinolones, it does interact indirectly with Mg^2+^ through hydrogen bond network intermediated by waters, extending the interaction to Asp87 [69]. These two residues, as well as Lys447, are associated with the quinolone’s resistance [69,70,71].

The docking simulation regarding the best binding poses of quinolones on Topo IV (*S. pneumoniae*, PDBID: 8QMB) (Figure 15B, Supplementary Figure S2 and Table S1) showed scoring values from 66.7191 (moxifloxacin) to 124.47 (gatifloxacin). The cocrystallized ligand (delafloxacin) displayed a ChemPLP score of 102.461. The binding interaction analysis revealed similar interactions to the ones previously reported in DNA Gyrase:

(1) The quinolone nucleus is sandwiched between two deoxyadenosines (Ade1 and Ade5) and binds through π-π interactions with two deoxythymidines (Thy4 and Thy15)—in pink in the figure.
(2) In an analogous binding, the carbonyl and the carboxylate groups from position 3 and 4 also may perform electrostatic interactions with Mg^2+^—grey dotted line.
(3) The carboxylate group from position 3 may interact through electrostatic interactions with Arg117 (Arg1117 in PDB file) from chain B and through hydrogen bond with Ser1079 from chain A—orange dotted line.
(4) The fluorine group at position 6 may interact with the carbonyl group of Thy 4 and15 through halogen bonds—cyan dotted line.
(5) Additional interactions with chain A may involve Leu412, Asp435 and Arg456.

The comparison of the docking scores and poses with the half-maximum inhibitory concentration values (IC_50_) from each enzyme (Supplementary Table S2) showed some agreement in *E. coli* DNA gyrase evaluation. The first-generation quinolones, nalidixic and oxolinic acids, were found at the bottom of the rank with 83.1213 and 88.2458 ChemPLP scores, fewer points higher than the last positioned quinolone premafloxacin (ChemPLP: 81.8371), which may suffered penalization due to clash and unfavorable bonds. Nevertheless, the highest ranked compounds trovafloxacin (118.133), both isomers of tosufloxacin (R:111.253; S:106,57) and gatifloxacin (109.519) corresponded to the inhibition values reported in the literature. On the other hand, the results found for *S. pneumoniae* showed some issues regarding the capacity of ChemPLP to predict quinolone activities, especially when we analyzed the last-ranked compound, moxifloxacin (ChemPLP: 66.7191), and compared its IC_50_ value on *S. pneumoniae* decatenation activity (15.047 µM), whereas both first-generation quinolones ranked 17th and 18th (oxolinic and nalidixic acids, respectively). One potential reason for these findings may be related to the difficulty for scoring functions to predict correctly the interaction energies between ligand, receptor and their environment. Both enzymes rely heavily on water networks and metallic interactions, whereas this validated protocol has not considered the water role. Another potential reason that should be considered is the size of the crystallographic binding site, which is suitable for delafloxcin but could be insufficient for bulkier compounds such as moxifloxacin. Our findings converge with the ones reported in the literature by Lupala and co-workers, on which the authors docked 15 quinolones into topo IV-DNA cleaved complexes from *S. pneumoniae* and *A. baumanii* and observed that their poses did not differ from the crystal poses. Moreover, they reported three regions of interactions for hydrophobic interactions (the fused rings intercalation area, the region at N1 substituents and the region close to N7 substituents). Finally, the authors concluded that Arg117 and Ser79 are important for the activity and resistance, respectively, while Asp83 may act as a residue involved in the early recognition step. Also, they observed that the bound orientation of the ligands was not altered by the presence or absence of Mg^2+^ and suggested that magnesium may be already bound to quinolone before binding to the enzyme [72].

### 4.3. Structure–Activity and Structure–Toxicity Relationships of Quinolones

#### 4.3.1. Structure–Activity Relationships

**Figure 16:**
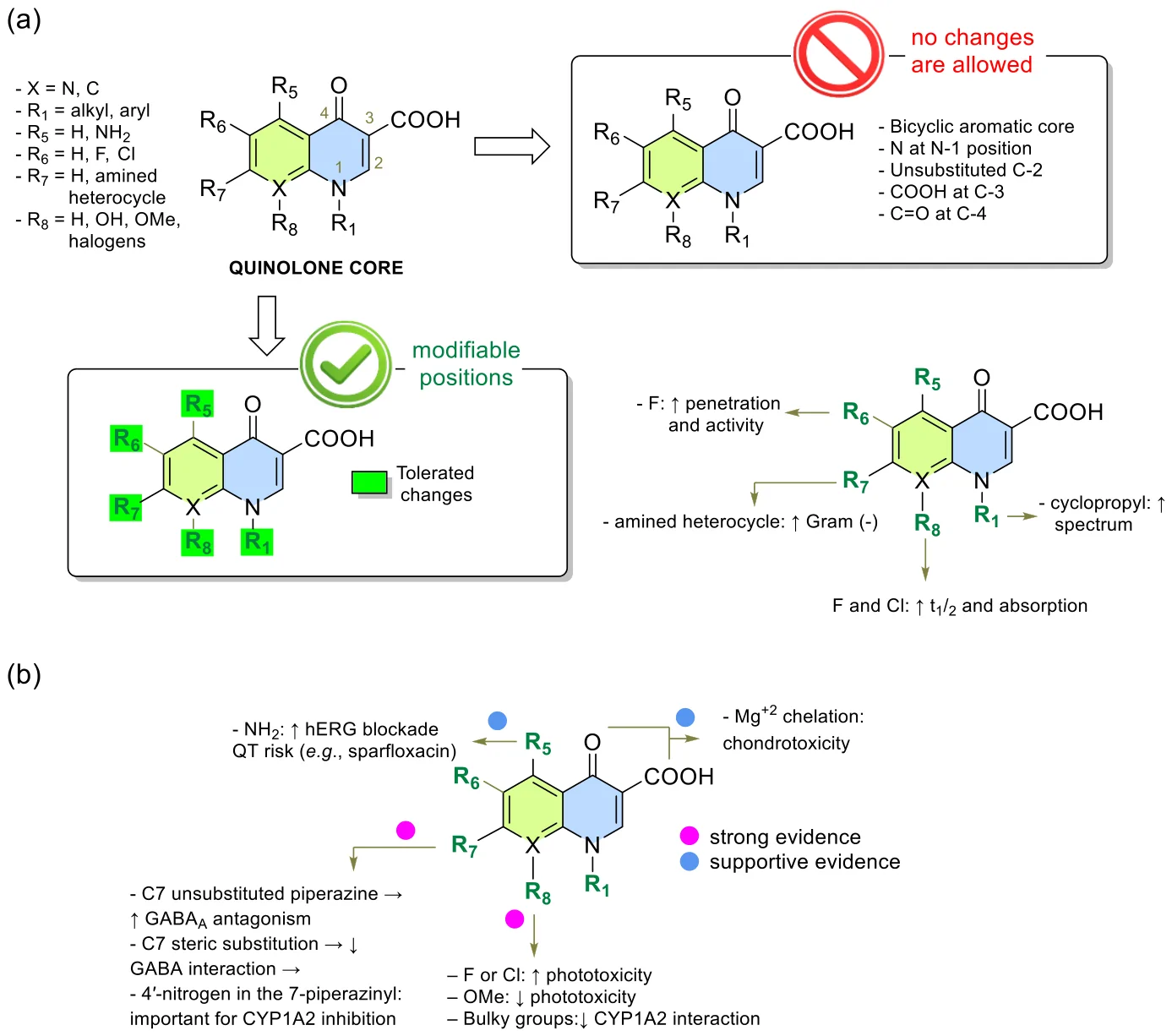
Quinolone pharmacophore highlighting the principal positions involved in structure–activity relationships (**a**) and structure–toxicity relationships (**b**).

The structure–activity relationship (SAR) identified for the quinolones was proposed by several researchers and the main findings were compiled here [15,16,17,18,65,71,73]. Comparative data of the half-maximal inhibitory concentration value (IC_50_) towards DNA Gyrase and Topo IV are reported at Supplementary Table S2. First, it is necessary to identify, within the overall structure of quinolones, the essential core (pharmacophoric group) and the fragments that have been added (or replaced) in order to improve the PD and/or PK profile of these drugs. As we were able to observe from the molecular docking performed (Figure 15), there are three important chemical characteristics, which are essential for the antimicrobial properties of quinolones (Figure 16a). One is the presence of a bicyclic aromatic system, of the 1-quinolone type, crucial for acting like an intercalating agent and interacting at the DNA level. Another is the presence of a hydrogen atom at the Cα of the enamine group (N-CH=C) from pyridone ring. Finally, the two vicinal carboxylic acid and carbonyl groups at C3 and C4, respectively. This set of characteristics forms the pharmacophoric group of quinolones and cannot be modified without completely losing antimicrobial activity. Therefore, the modifications can be performed at R1, R5, R6, R7, and R8, as we depicted in Figure 16a. This figure shows the main chemical characteristics and modifications that impact the PD and PK of quinolones and can be summarized as follows:

**Position 1**: Modifications in N1 can alter the antibacterial potency and PK. Introduction of a cyclopropyl group at N1, as observed in ciprofloxacin, gatifloxacin, and moxifloxacin, has been associated with favorable antibacterial activity and pharmacokinetic properties), while other fragments provided no benefit or even caused serious adverse effects (Temafloxacin [74]).
**Position 2**: This position is the only one in the quinolone scaffold that cannot be modified. The modifications made led to compounds with a much lower antibacterial profile.
**Positions 3 and 4:** The two carbonyls at C3 and C4 (3-carboxylate and 4-keto groups) are fundamental for binding with Gyrase and Topo IV, as they form electrostatic interactions with divalent metals such as Mg^+2^. The carboxylate group at C3 can form electrostatic interactions with Arg and Ser residues. Another carboxylate contribution is the increased solubility, influencing physicochemical properties and impacting PD and PK.
**C5 Position**: Modifications at C5 led to drugs with greater activity against Gram-positive.
**C6 Position**: The introduction of F results in greater potency and antibacterial spectrum: Fluorine interacts with Gyrase and Topo IV enzymes through halogen bonds with the thymine groups.
**C7 Position**: The basic ring at the C7 position forms a zwitterion with COOH-C3, increasing penetration into bacterial cells, resulting in increased activity. It also resulted in increased tissue penetration, improving PK properties such as higher half-life (t_1/2_) or volume of distribution.
**C8 Position**: The introduction of F led to an increase in absorption and half-life [75].

#### 4.3.2. Structure–Toxicity Relationships

Beyond antibacterial potency, structural modifications of the quinolone scaffold also markedly influence adverse-effect profiles (Figure 16b). At C5, an amino group can enhance hERG/KCNH_2_ K^+^ channel blockade, an effect associated with delayed ventricular repolarization, QT/QTc prolongation, and increased cardiotoxic risk. Sparfloxacin, a representative 5-NH_2_ quinolone, displays substantially greater hERG inhibition than norfloxacin or lomefloxacin, and its C5-NH_2_ group has been identified as an important structural determinant of this effect [76]. However, a C5 amino group is not required for hERG blockade. Moxifloxacin, which lacks C5-NH_2_, also inhibits hERG; mechanistic studies showed preferential block of the activated channel and markedly reduced inhibition following mutation of Tyr652, consistent with direct interaction within the S6-lined inner cavity [77].

At C7, an unsubstituted piperazinyl group is associated with increased proconvulsant liability. Enoxacin, norfloxacin, ciprofloxacin, and pipemidic acid, all bearing an unsubstituted C7 piperazine, strongly inhibited [3H]muscimol binding in the presence of biphenylacetic acid (metabolite of fenbufen) and produced marked epileptogenic effects in mice, consistent with antagonism of inhibitory GABAA signaling [78]. Increasing substitution within the C7 piperazinyl moiety can attenuate proconvulsant liability: in the BPAA-sensitized mouse model, ofloxacin and sparfloxacin (AT-4140), both bearing methyl-substituted piperazines, did not induce convulsions even at 100 mg/kg, whereas enoxacin, norfloxacin, ciprofloxacin, and pipemidic acid, which contain unsubstituted C7 piperazines, induced clonic convulsions at doses ≥ 6.25 mg/kg [78]. Consistent with this lower proconvulsant potential, ofloxacin was substantially less potent than ciprofloxacin at inhibiting GABA-evoked currents, with IC_50_ values of >1 mM versus approximately 100 µM in the absence of BPAA and 0.3 µM versus 0.03 µM in its presence [79]. However, methyl substitution is not universally protective: lomefloxacin, despite bearing a 3-methylpiperazine at C7, induced clonic convulsions at doses ≥6.25 mg/kg in the same BPAA-sensitized model, indicating that the position and overall pattern of C7 substitution, rather than methylation or steric bulk alone, influence proconvulsant liability [78].

C7 substitution also modulates CYP1A2 inhibition: an unsubstituted 7-piperazine generally increases inhibitory potency, whereas N-alkylation decreases it [80]. For enoxacin, proton-NMR measurements detected strong interaction to the CYP1A2 heme and implicated the distal 4′-piperazinyl nitrogen, consistent with donation of its lone pair toward the heme iron [81].

C8 is the dominant structural determinant of phototoxicity. Halogen substitution, exemplified by 8-F in lomefloxacin, fleroxacin, and sparfloxacin and 8-Cl in besifloxacin, delafloxacin and sitafloxacin, increases photoreactivity, whereas 8-OCH_3_, exemplified by moxifloxacin, gatifloxacin, balofloxacin, nemonoxacin and lascufloxacin markedly reduces it [82]. Upon ultraviolet excitation, the quinolone accesses excited states that can undergo electron-transfer chemistry and generate reactive oxygen species. In 8-halogenated derivatives, internal charge transfer additionally favors heterolytic C8–X photofragmentation, producing highly electrophilic aryl cations capable of reacting with nucleophiles and DNA [83]. Conversely, substitution at C-8 with a methoxy group prevents the photodehalogenation pathway characteristic of 8-halogenated fluoroquinolones, markedly increasing photostability and decreasing oxidative DNA damage and phototoxicity. Under UVA irradiation, the 8-methoxy quinolone BAY 12-8039 generated more than fourfold less 8-oxo-dG than the corresponding 8-chloro analogue BAY Y3118 in ARL-18 cells [84].

Finally, the 3-carboxylate/4-keto chelating motif has important implications for antibacterial activity, pharmacokinetics, and toxicity. This moiety coordinates Mg^2+^ through the water–metal-ion bridge that stabilizes quinolone binding to bacterial topoisomerase–DNA complexes (as previously described in the docking analysis). It can also chelate multivalent cations present in the gastrointestinal tract, thereby reducing quinolone absorption and oral bioavailability. Additionally, the chelation of Mg^2+^ in cartilage has been proposed as a potential contributing mechanism to quinolone-associated chondrotoxicity, although its precise role remains incompletely understood [85].

## 5. Pharmacokinetics

Numerous papers describing the pharmacokinetic parameters of quinolones are available, reporting studies with different animals’ species due to their wide application in both veterinary and human medicine [86,87]. This paper will focus mainly on *in vitro* data and quinolone’s pharmacokinetic parameters estimated in humans. The main part of references about which will be discussed can be found in the respective Supplementary Materials as reported in the text.

In general, quinolones have good bioavailability, high tissue distribution, are eliminated by both metabolism and renal excretion and an intermediate half-life. Quinolones are substrate to membrane influx transporters, which helps to explain their high tissue penetration, and efflux transporters such as P-glycoprotein (P-gp). The molecular alterations that characterize the different quinolone generations broadened the spectrum of susceptible bacteria also affected the pharmacokinetics, as presented following only for the quinolones that are currently in the market for human use, except when indicated otherwise.

### 5.1. Pharmacokinetics of First-Generation Quinolones

NA, the template of first-generation quinolones, is no longer used in human medicine. It was indicated for the treatment of urinary tract infections by Gram-negative bacteria, primarily Enterobacteriaceae (except *Pseudomonas* species) (Supplementary Table S3). The drug presented high bioavailability (96%), and protein binding (92–97%), low volume of distribution, and it was eliminated mainly by metabolism (>95%) (Supplementary Table S4). The elimination half-life was first reported in the range of 1–2.5 h; however, Cuisinaud et al., using more sensitive bioanalytical method, indicated that the elimination half-life would be longer (6–7 h) [88].

### 5.2. Pharmacokinetics of Second-Generation Quinolones

The second generation of quinolones includes NOR, CIP and OFLX. Lomefloxacin also belonged to this group but it was withdrawn from the market in 2008 [89]. These quinolones introduced a fluorine atom (fluoroquinolones) expanding the spectrum of activity to include *Pseudomonas aeruginosa* and some Gram-positive bacteria (Supplementary Table S3), and increased tissue penetration. They are highly active against Gram-negative bacteria and their indications also include besides urinary tract infections, pulmonary infections and other soft tissue infections (Supplementary Table S3).

NOR was the first fluoroquinolone to be introduced in the market (Supplementary Table S4) [89]. It shows lower protein binding (15%) compared to NA, a smaller bioavailability (30–50%), with a high volume of distribution (1.7 L/kg). It is eliminated by three equally important pathways: renal, biliary and metabolism, forming six metabolites less active than the original compound, with a half-life of 3 h.

CIP presents a rapid absorption with a bioavailability around 70–80%, low protein binding (25%), and high volume of distribution around 2.0–3.5 L/kg (Supplementary Table S4). It is eliminated via renal excretion and metabolism, forming four metabolites (desethyleneciprofloxacin, sulfo-ciprofloxacin, oxo-ciprofloxacin and N-acetylciprofloxacin) less potent than the parent drug [89], with a half-life around 5 h.

OFLX shows an almost complete bioavailability (80–99%) following oral dosing, low plasma protein binding (25%) leading to a high tissue distribution (Volume of distribution = 2.4–3.5 L/kg) (Supplementary Table S4). Different from ciprofloxacin, it is excreted mostly in the urine (80%), with small fraction being metabolized (<5%), and a longer half-life (5.0–7.5 h) than the other second-generation quinolones.

### 5.3. Pharmacokinetics of Third Generation Quinolones

The third-generation quinolones include LEV, TMFX and SPFX. Due to important side effects such as increased risk of dysglycemia, low blood sugar, hemolytic anemia, kidney and liver dysfunction, allergic reactions, phototoxicity and QT prolongation, some were withdrawn from the market, including TMFX (1992) and SPFX (2001) [89]. The third-generation quinolones improved and extended the activity against Gram-positive bacteria (particularly *Streptococcus pneumoniae*) and atypical pathogens (*Mycoplasma pneumoniae*, *Chlamydia pneumoniae*), while retaining broad Gram-negative coverage (Supplementary Table S3). In general, they have similar indications as the second generation.

LEV pharmacokinetics is characterized by rapid and complete oral absorption (bioavailability ~100%) (Supplementary Table S4). The drug is moderately bound to plasma proteins (24 to 38%) and shows a moderate volume of distribution (1.1 to 1.5 L/kg). It is eliminated mainly by urinary excretion (>87%), with a half-life of 4 to 7 h.

PZFX is used as *i.v.* infusion, with a small volume of distribution (0.62 ± 0.11 L/kg), a high clearance via renal excretion, in the range of 13.3 to 21.7 L/h in average, and a short half-life (2.99 ± 0.5 h) (Supplementary Table S4).

TFLX is used orally, having an intermediate protein binding (~37%), high clearance (12.1 ± 0.6 L/h) and short half-life (3.5 ± 0.2 h) (Supplementary Table S4).

### 5.4. Pharmacokinetics of Fourth Generation Quinolones

The fourth generation of quinolones includes BSF, GMFX, MXF, GAT, TRO, STFX and LSFX. Trovafloxacin TRO was withdrawn from the market in 2001 because of the risk of liver failure among the patients [89], and GMFX received an FDA warning in 2016 due to high rates of rash in specific populations and risks of tendon ruptures and tendonitis, especially in patients over 60. The drug was withdrawn from the market by the pharmaceutical company in 2024. GAT, is still commercialized in some countries only for topical ocular use. The fourth generation retains the broad Gram-negative and Gram-positive activity of the third generation but gains significant activity against anaerobic bacteria which is fundamental for treating mixed aerobic and anaerobic infections such as intra-abdominal infections (Supplementary Table S3).

BSF, a fourth-generation quinolone was the first topical chlorofluoroquinolone (2009) for treatment of bacterial conjunctivitis. It has never been used as a systemic antimicrobial, only in topical applications, with high concentrations been found in tears and conjunctiva after a single topical dose [90].

MXF is rapidly and almost completely absorbed following oral dosing (86–90% bioavailability). It presents a higher protein binding (30–50%) than LEV, presenting a high volume of distribution (1.7–2.7 L/kg). It is eliminated mainly by metabolism forming two inactive metabolites (sulfo-compounds—M1; glucuronide—M2) [89], with a longer half-life ranging from 11 to 15 h (Supplementary Table S4).

LSFX presents an intermediate protein binding (74%), a high volume of distribution (2.46–4.11 L/kg) and a half-life longer than moxifloxacin (15.6–18.2 h) (Supplementary Table S4).

STFX shows as intermediate protein binding (50%), smaller volume of distribution (1.46–2.57 L/kg) than LSFX, higher clearance (up to 19.7 L/h) and smaller half-life (up to 7h) (Supplementary Table S4).

The indication of quinolones for the treatment of urinary tract and pulmonary tract infections (Supplementary Table S3), besides other types of infections according to generations, is related to their penetration into different tissues. Quinolones are reported to have high tissue penetration factor (PRu), determined by the ratio between exposure to pharmacologically active (unbound to proteins) concentrations in tissue interstitial fluid over exposure to unbound concentrations in plasma (PRu = AUC_free,tissue_/AUC_free,plasma_). When this ratio is equal to units, it indicates that antimicrobial tissue penetration is mainly driven by diffusion, with no interference of membrane transporters. If the ratio is bigger than one, influx transporters play a role on drug tissue distribution and, if smaller than unit, efflux transporters reduce drug penetration in the tissue under investigation. Because determination of unbound concentrations in tissues requires sophisticated techniques such as microdialysis, only few studies have been conducted to determine quinolones tissue penetration in humans. Supplementary Table S4 shows the influx/efflux transporters already reported and Supplementary Table S5 shows the tissue penetration factor of some quinolones in different human tissues.

One can observe that most of the penetration studies were conducted in muscle and subcutaneous adipose tissue, which are easily accessed in humans. In these tissues, quinolones in general show a PRu close to unit. However, these tissues do not express the influx/efflux transporters reported for quinolones. In animal models, tissue penetration in other organs have been reported, showing influx/efflux transporters to lung (CIP PRu = 1.2; GAT PRu = 1.08; LEV PRu = 0.69) [91,92] and prostate (LEV PRu = 0.68; MXF PRu = 1.24; ciprofloxacin CIP PRu = 1.5) [93,94,95], for instance, and brain efflux of all quinolones [96].

### 5.5. Pharmacokinetic/Pharmacodynamic Index (PK/PD Index) and Posology

The relationship between pharmacokinetics (PK) and pharmacodynamics (PD) is used in clinical practice to adjust dosing regimens for antimicrobials. The PK/PD index relates a pharmacokinetic parameter (peak plasma concentration—Cmax; area under the plasma concentration–time curve; period of time with plasma concentrations above certain level—T) and the PD parameter minimum inhibitory concentration (MIC). PK/PD indices are used to classify antimicrobials in time-dependent killing and concentration-dependent killing. The antimicrobial is said to have time-dependent killing (fT > MIC) if the antimicrobial activity depends on the percentage of time that free plasma concentrations remain above the MIC in a 24 h or dosing interval period. On the other hand, the antimicrobial is said to have a concentration-dependent effect when the ratio of free plasma concentration over MIC (fC_max_/MIC) or the free plasma exposure over MIC (fAUC/MIC) are best related to antimicrobial efficacy. Quinolones are concentration-dependent antimicrobials, with the fAUC_0-24_/MIC been more strongly related to successful outcome and used to select dosing regimens and the PK/PD index fCmax/MIC being considered a complementary PK/PD index associated with antibacterial efficacy and resistance suppression [97].

Supplementary Table S5 shows the PK/PD index of some quinolones. One can observe that the target value of fAUC_0-24_/MIC is similar for quinolones of different generations and always higher for infections caused by Gram-negative bacteria (between 87 and 125) than by Gram-positive bacteria (between 30 and 50) [98]. The secondary fC_max_/MIC is reported for some quinolones and ranges around 8–12. Consequently, based on bacterial characteristics and MIC, quinolones dosing regimens are selected to reach the PK/PD index targets.

## 6. Quinolone Adverse Effects and Toxicity

Quinolones are associated with severe adverse effects and, for this reason, some received FDA and EMA warnings over the years or were withdrawn from the market by regulatory authorities or manufacturers. The most common adverse effects of the quinolones are gastrointestinal effects (nausea, vomiting, abdominal discomfort and pain, anorexia, and in some cases, diarrhea) and arthralgia (or joint pain), which are associated with the structural feature of the quinolone pharmacophore [71,85]. Consequently, this class is limited for prescription to pediatric patients [71]. Although controversial reports about other adverse effects can be found in the literature, with some mild but frequent, and others rare but severe, this antimicrobials are mostly associated with aortic aneurysm and aortic dissection, tendinopathy/tendon injury (particularly to the Achilles tendon), retinal detachment, peripheral neuropathy, neuropsychiatric toxicity, seizures, exacerbation of myasthenia gravis, cutaneous side-effects, phototoxicity, *Clostridium difficile* infection, fatal arrhythmia due to QT prolongation, dysglycemia/hypoglycemia, hepatotoxicity, genotoxicity, severe hemolytic-uremic syndrome, and acute renal failure [16,99,100]. Due to their potential severe side effects, regulatory agencies recommend the use of quinolones only for severe infections or when first-line antimicrobials fail [101].

As mentioned, some quinolones side effects are related to the antimicrobial general pharmacophore, been reported for all quinolones with different incidence (gastrointestinal discomfort or arthralgia). The presence of carboxylate function that prone quinolone to form complexes with divalent and trivalent metal ions including iron, magnesium, and calcium is, therefore, an unavoidable adverse effect. Most of the other unwanted effects of quinolones are dependent on their substituents and, consequently, specific to a particular generation and/or agent [16,102].

Some of the most frequent or severe adverse effects related to quinolones are presented below. For more information on quinolone toxicity, one can search review papers in the literature that detail adverse effects and their mechanisms [16,71,99,100].

The aortic aneurysms/dissections are infrequent but severe adverse effect due to the dangerous bleeding that threatens the patient’s life. This adverse effect is more common with CIP, LEV, and MXF. The proposed mechanism claims that quinolones upregulate cell matrix metalloproteinases (MMPs), reducing collagen fibrils (types I and III), interfering with extracellular matrix (collagen and elastic fibers) integrity in the aortas. This process impairs biomechanical strength triggering progressive aortic degradation until dissection or rupture. Furthermore, FQs strongly chelate the iron needed by the two essential iron-dependent enzymes in synthesizing collagen (prolyl 4-hydroxylase and lysyl hydroxylase) [16].

Other severe adverse effect observed with the use of quinolones, especially LEV, is tendinitis, which in 50% of cases is bilateral, and tendon rupture. Tendinitis is explained by the high quinolone’s affinity to connective tissues such as bones and cartilage. The theories used to explain the tendinopathy are based on ischemic influences, matrix-degrading phenomenon, and toxic changes caused by quinolones, besides the upregulation of MMPs. Consequently, most collagen fibrils found in Achilles’ tendons will be reduced. As a result, the tendon cells will suffer degenerative events (organelle dilatation, vacuole formation, and apoptosis), leading to rupture [16].

A QT prolongation with ventricular arrhythmia and eventual torsades de pointes are another quinolone’s adverse effects that can lead to patient’s sudden death [16,100]. The QT interval in an electrocardiogram represents the time it takes for the heart’s ventricles to contract and then relax. It indicates ventricular depolarization and repolarization, with normal durations typically between 350 and 440ms. The prolongation of the QT interval is mainly based on the increased repolarization duration through the blockade of the cardiac K^+^ channel (human ether-a-go-go gene, hERG) and can be observed with GAT, GPFX, LEV, MXF, GMFX, and SPFX. Cardiotoxicity, including QT prolongation led to withdrawn of GPFX and SPFX from the market. The MFX-induced QT interval prolongation could lead to torsades de pointes, which is a potentially fatal polymorphic ventricular tachyarrhythmia characterized by the twisting of points on an electrocardiogram.

Quinolone treatment can lead to alterations in glucose metabolism, with increased risk of dysglycemia or severe hypoglycemia, which are reversible upon discontinuation of treatment. The hypoglycemia associated with LEV can be severe, persistent, and life-threatening. GAT was withdrawn from the market due to severe and fatal hypoglycemia. Although the underlying mechanisms of dysglycemia are unknown, it is hypothesized that quinolones could show a sulfonylurea-like effect by blocking the secretagogues ATP-sensitive K^+^ channels responsible for the insulin release from the pancreas, depolarizing the pancreatic β-cell membrane, allowing the entry of calcium and the release of insulin, leading to important hypoglycemia. Other hypothesis involves the inhibition of CYP450 enzymes (CYP2C9, CYP2C8, and CYP3A4) responsible for antidiabetic’s drug metabolism, leading to higher blood levels of these drugs and decreased sugar levels. This last hypothesis, however, is only acceptable if the patients use antidiabetic drugs during quinolone treatments, which was not always the case of the severe hypoglycemia reported for these antimicrobials [16,100].

Treatments with quinolones, especially CIP, LEV, MXF, and OFLX can also be associated with peripheral nerve or acute central nervous system (CNS) dysfunction. The CNS adverse effects are the second most frequent after gastrointestinal-related ones, ranging from mild effect such as confusion, irritability, and insomnia, to severe ones such as encephalopathy, seizures, suicidal depression, catatonia, psychosis, and mania [100]. The underlying mechanisms of neurotoxicity are the inhibition of GABA (gamma-aminobutyric acid) receptors, where 7-piperazine or pyrrolidinyl without substituents may be essential in interacting with these receptors, and NMDA (N-methyl-D-aspartate) receptors. Quinolones with unsubstituted heterocycles in position C7 seem more associated with central adverse effects [16,100].

*Clostridioides difficile* infection is currently one of the most frequent reported adverse effects associate with quinolone treatment [100]. Due to their broad-spectrum of activity and the fact that quinolones such as CIP and MXF are eliminated by biliary excretion and can reach high concentrations in the feces, these quinolones may alter the colon microbiota, leading to disturbance of gut microbiota balance and *C. difficile* infection, characterized by diarrhea and colitis.

According to Rusu et al. (2023) [16], the quinolones withdrawn from therapy due to adverse effects were, from the second generation: fleroxacin in 1990 (CNS effects, phototoxicity), temafloxacin in 1992 (hemolytic-uremic syndrome), and TFLX in 2006 (hematological and other adverse effects). Other withdrawn quinolones include: grepafloxacin in 1999 (QT prolongation, fatal cardiotoxicity, gastrointestinal toxicity), clinafloxacin in 1999 (hypoglycemia, phototoxicity), trovafloxacin in 2000 (hepatotoxicity), SPFX in 2001 (QT prolongation and phototoxicity), alatrofloxacin in 2006 (seizures, thrombocytopenia, hepatotoxicity), GAT in 2006 (increased risk of dysglycemia), and GMFX in 2009 (rash erythematous).

## 7. Mechanism of Resistance of Quinolones

The widespread clinical and veterinary use of quinolones has been followed by the occurrence of resistant bacterial populations, compromising the efficacy of this important antimicrobial class and leading to a change in the direction regarding the usage of this therapeutic class (antibiotic stewardship) [71,103]. The World Health Organization Global Antimicrobial Resistance and Use Surveillance System (GLASS) 2025 report highlights that resistance to fluoroquinolones remains a major global health interest. High levels of ciprofloxacin resistance were observed in Neisseria gonorrhoeae (75.0%), while resistance in urinary tract infections caused by *E. coli* and *Klebsiella pneumoniae* exceeded 30% globally. In bloodstream infections, ciprofloxacin resistance was also substantial, reaching approximately 40% in *E. coli* and around 50% in *K. pneumoniae*. Additionally, resistance to ciprofloxacin was reported in non-typhoidal *Salmonella* spp. (18.0%), and fluoroquinolone resistance in *Shigella* spp. approached 30% globally, with higher levels in some regions (Southeast Asia, 75.5%). These findings emphasize the widespread distribution and clinical significance of fluoroquinolone resistance across multiple bacterial pathogens [104]. A study conducted by Ajulo & Awosile found correlation between fluoroquinolones consumption and antimicrobial resistance towards bloodstream infections caused by *E. coli* and *K. pneumoniae* [105].

Even though the development of resistance has traditionally been associated with mutations in the genes encoding DNA gyrase and Topo IV, current evidence shows that quinolone resistance results from the combined action of several mechanisms. These include target-site alterations, reduced intracellular drug concentration due to decreased permeability or increased efflux (MDR efflux pumps) and acquisition of transferable resistance elements [26,65,106,107,108,109,110]. Moreover, it has been suggested that quinolones usage with sublethal doses or sub-MIC exposure and incomplete treatment may lead to the development of resistance towards non-quinolones antibiotics [65,111]. Therefore, understanding quinolone resistance mechanisms is of utmost relevance for medicinal chemists, pharmacists, clinicians, and students from several disciplines.

The typical and nonetheless most relevant mechanism associated with quinolone resistance is the accumulation of mutations in the genes encoding DNA gyrase (gyrA and gyrB) and Topo IV (parC and parE) [110]. In Gram-negative bacteria, mutations in gyrA are usually the most relevant, whereas in Gram-positive bacteria mutations in parC are often more important [110]. These mutations are commonly located in quinolone resistance-determining regions (QRDRs), which are situated between amino acids 67 and 106 in gyrA (*Escherichia coli* numbering), most frequently associated with Ser83 and Asp87 in *E. coli*, and between 63 and 102 in parC, especially regarding the residues from Ser79 and Asp83 [65,110]. Nonetheless, QRDRs in gyrB and parE have been found, with the most common mutations found in gyrB D426N and L447E (*E. coli* numbering) [65]. Substitutions of the amino acids in QRDRs may interfere with the water–metal ion bridge, consequently diminishing quinolone binding to the enzyme–DNA complex while preserving essential topoisomerase activity [110]. Some studies highlighted that the most common mutation occurs at serine residues, which appear to have low effect on catalytic activity. On the other hand, the mutations at the acidic residue may reduce enzyme activity from 5 to 10 fold [71].

In addition to target-site alterations, decreased intracellular accumulation of quinolones is a major contributor to resistance. This may result from reduced permeability, especially through changes in outer membrane porins in Gram-negative bacteria, or from increased activity of multidrug efflux pumps. Several efflux pump families, including RND, MFS, MATE, and ABC transporters, can participate in quinolone resistance [110]. Efflux-mediated resistance frequently produces low to moderate increases in MIC, but it can contribute to clinically relevant resistance when combined with QRDR mutations or other mechanisms. Because many efflux pumps have broad substrate profiles, their overexpression may also promote multidrug resistance and cross-selection by unrelated antibiotics, disinfectants, or toxic compounds [65,112].

Transferable mechanisms of quinolone resistance (TMQR) have substantially changed the epidemiological understanding of quinolone resistance. Although quinolones are synthetic antimicrobials and resistance was initially expected to arise mainly through chromosomal mutations, several mobile genetic determinants are now recognized (plasmids, integrons, transposons, genomic islands, bacteriophages, or chromosomal regions acquired through horizontal gene transfer). TMQR are usually classified into three main groups: target protection, drug modification, and transferable efflux [26,109].

Quinolone exposure, particularly at sublethal concentrations, plays a critical role in promoting resistance not only to quinolones but also to non-quinolone antibiotics through the activation of global stress responses and evolutionary pathways. Quinolone-induced DNA damage triggers the SOS response, leading to increased mutagenesis, recombination, and genomic plasticity, which collectively enhance the probability of resistance emergence across multiple antibiotic classes [65]. This concept is further reinforced by recent findings demonstrating that subinhibitory antibiotic concentrations can stimulate horizontal gene transfer, enrich resistance genes within bacterial populations, and increase the formation of persister cells, all of which contribute to multidrug resistance development [111].

## 8. Drug-like Property Profiling of Quinolones

**Figure 17:**
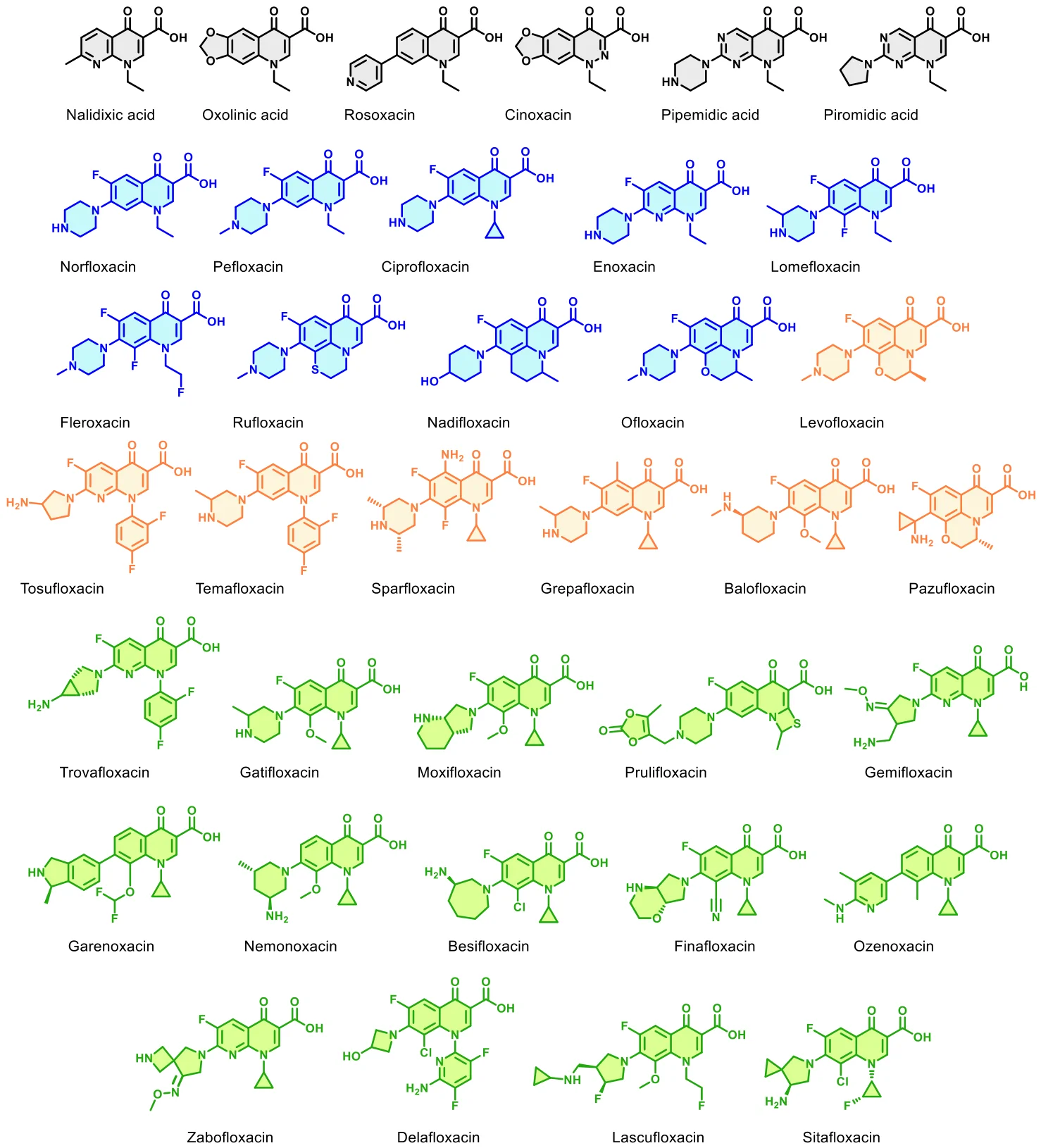
Structural evolution of quinolone antibacterial agents. Structures of the quinolones included in the drug-like property analysis, arranged according to their generation. First-generation quinolones are shown in gray, second-generation compounds in blue, third-generation compounds in orange, and fourth-generation compounds in green. Progressive structural diversification is evident across the series, with later quinolones generally displaying larger and more complex peripheral substituents while retaining the conserved quinolone pharmacophore. Generation assignments follow a four-generation classification scheme; alternative classifications have been proposed for certain quinolones.

The structural diversification of quinolones over successive generations is readily apparent from comparison of their structures (Figure 17). Early quinolones are generally characterized by relatively small and compact scaffolds, whereas later compounds incorporate increasingly elaborate peripheral substituents, larger heterocyclic moieties, and a greater number of potentially rotatable bonds.

Drug-like molecular descriptors of 35 quinolones [20] were retrieved from SwissADME [113] (Supplementary Table S6) and subjected to principal component analysis (PCA) to obtain an exploratory overview of how the physicochemical profile of this antibacterial class evolved over time. The descriptors included molecular weight (MW), fraction of sp3-hybridized carbons (Csp^3^), number of rotatable bonds, hydrogen-bond acceptors (HBA), hydrogen-bond donors (HBD), topological polar surface area (TPSA), and consensus logP. Importantly, descriptors should be calculated for the neutral parent compound rather than for pazufloxacin mesylate or other salt forms, since inclusion of counterions may artificially inflate MW, HBA/HBD, TPSA, and related drug-like descriptors. The first two principal components explained 61.65% of the total variance, with PC1 and PC2 accounting for 42.23% and 19.42%, respectively.

**Figure 18:**
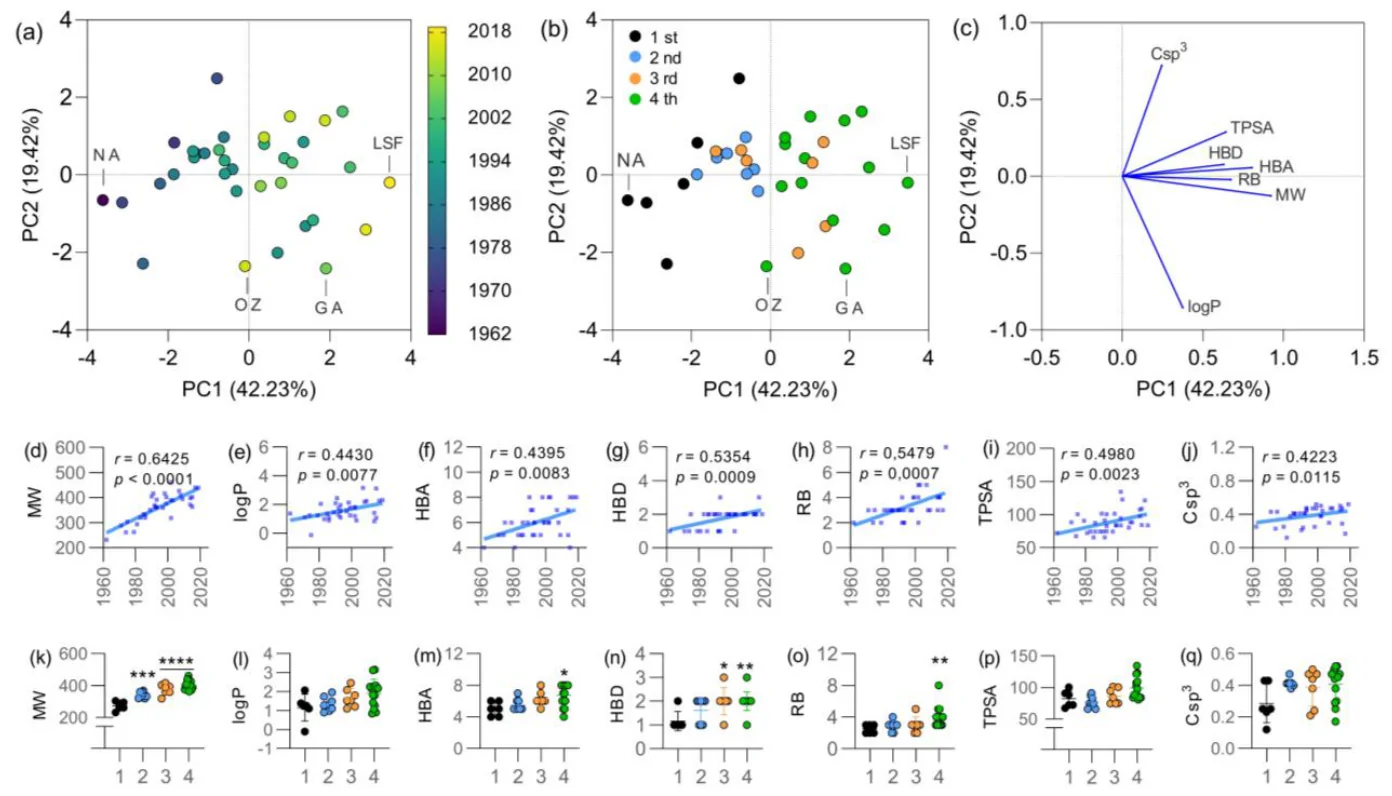
Evolution of drug-like properties across quinolone generations. Drug-like descriptors of 35 quinolones were retrieved from SwissADME and analyzed by principal component analysis. The descriptors included molecular weight (MW), lipophilicity (logP), hydrogen-bond acceptors (HBA), hydrogen-bond donors (HBD), rotatable bonds (RB), topological polar surface area (TPSA), and fraction of sp^3^-hybridized carbons (Csp^3^). Only ofloxacin (second generation) was included, as it shares identical molecular descriptors with levofloxacin (third generation). (**a**) PCA score plot colored by year of quinolone introduction. (**b**) PCA score plot colored by quinolone generation. (**c**) Loading plot showing descriptor contributions to the PCA space. (**d**–**j**) Correlations between year of introduction and individual descriptors; coefficients and *p* values are shown in each panel. Pearson correlation was used for MW and logP, whereas Spearman correlation was used for HBA, HBD, RB, TPSA, and Csp^3^. (**k**–**q**) Generation-wise comparisons of individual descriptors (mean ± standard deviation). ANOVA followed by Tukey’s test was used for MW and logP, whereas Kruskal–Wallis followed by Dunn’s test was used for HBA, HBD, RB, TPSA, and Csp^3^. Pearson’s correlation coefficients (r) were calculated for MW and logP, while Spearman’s rank correlation coefficients (r_s_) were calculated for the remaining variables. Asterisks indicate significant differences relative to first-generation quinolones: * *p* < 0.05, ** *p* < 0.01, *** *p* < 0.001, **** *p* < 0.0001. NA, LSF, OZ, and GA denote nalidixic acid, lascufloxacin, ozenoxacin, and garenoxacin, respectively, which mark representative extremes along PC1 and/or PC2. The figure was created using GraphPad Prism 10.

When compounds were colored according to year of introduction, a clear temporal organization was observed along the PCA space. Nalidixic acid (NA), the archetypal compound and historical starting point of the quinolone class, occupied the far-left region of the plot, whereas subsequently developed quinolones progressively expanded toward the central and right regions of the chemical space (Figure 18a). This distribution suggests that, from the initial nalidixic acid scaffold, successive quinolone generations explored a broader and more diversified physicochemical space, characterized by increasing values of several drug-like descriptors. A similar pattern emerged when compounds were colored according to quinolone generation: first-generation agents clustered predominantly in the left/central region, second- and third-generation quinolones occupied intermediate positions, and fourth-generation quinolones were enriched in the right region of the PCA plot (Figure 18b).

The loading plot indicated that the rightward displacement of newer quinolones was mainly associated with higher MW, HBA, HBD, TPSA, and, to a lesser extent, rotatable bonds, whereas Csp^3^ and logP contributed predominantly to separation along the vertical component (Figure 18c). These findings indicate that the evolution of the quinolone class was accompanied by a progressive increase in molecular size, hydrogen-bonding capacity, polarity, and structural diversification around the conserved quinolone core.

To further assess temporal trends in individual descriptors, each variable included in the PCA was correlated with the year of quinolone introduction. MW showed the strongest positive correlation with year of introduction (*r* = 0.6425, *p* < 0.0001; Figure 18d), indicating a marked increase in molecular size across successive generations. LogP also showed a significant positive correlation with year (*r* = 0.4430, *p* = 0.0077; Figure 18e). The highest logP values were found for ozenoxacin and garenoxacin, which also exhibited the lowest PC2 scores, indicating that these compounds represent the most lipophilic quinolones in the dataset and occupy the lower region of the PCA space. Similarly, HBA (*r* = 0.4395, *p* = 0.0083; Figure 18f), HBD (*r* = 0.5354, *p* = 0.0009; Figure 18g), and TPSA (*r* = 0.4980, *p* = 0.0023; Figure 18i) increased significantly over time, supporting the notion that later quinolones acquired enhanced hydrogen-bonding and polar surface features. The number of rotatable bonds was also positively correlated with the year of introduction (*r* = 0.5479, *p* = 0.0007; Figure 18h), indicating that more recently introduced quinolones tend to incorporate more conformationally flexible peripheral substituents while retaining the rigid quinolone pharmacophore. In our dataset, lascufloxacin displayed the highest number of rotatable bonds, which can be mainly attributed to its structurally elaborated substitution pattern, including a 2-fluoroethyl substituent at N-1 and a bulky C-7 fluoropyrrolidinyl substituent bearing a (cyclopropylamino)methyl moiety. Thus, although the quinolone core remains intrinsically rigid and largely planar, the progressive introduction of larger peripheral groups appears to have increased conformational flexibility in newer analogues. This trend is also visually evident in Figure 17, in which later quinolones generally display larger and more structurally elaborate substituents surrounding the conserved bicyclic core. Finally, Csp^3^ also showed a significant positive correlation with year of introduction (*r* = 0.4223, *p* = 0.0115; Figure 18j), suggesting that newer quinolones display a modest but measurable increase in three-dimensional character, likely due to the incorporation of non-planar substituents appended to the otherwise planar bicyclic quinolone core.

To complement the year-based correlation analysis, descriptor values were compared across quinolone generations to determine whether continuous temporal trends were also reflected in discrete generation-level physicochemical shifts. Molecular weight increased significantly in second- (*p* = 0.0003), third- (*p* < 0.0001), and fourth-generation quinolones (*p* < 0.0001) compared with first-generation compounds (Figure 18k). In contrast, logP did not differ significantly across generations (Figure 18l). HBA values were significantly higher in fourth-generation quinolones relative to first-generation compounds (*p* = 0.0408; Figure 18m). Similarly, HBD increased significantly in third- (*p* = 0.0330) and fourth-generation quinolones (*p* = 0.0073) compared with the first generation (Figure 18n). The number of rotatable bonds also increased across generations, reaching statistical significance in fourth-generation quinolones relative to first-generation compounds (*p* = 0.0082; Figure 18o). By contrast, TPSA did not differ significantly among second-, third-, or fourth-generation quinolones compared with the first generation (Figure 18p). Likewise, Csp3 values showed no significant generation-dependent differences, although considerable within-generation variability was observed (Figure 18q). Overall, generation-wise comparisons confirmed significant increases in molecular weight, hydrogen-bonding capacity, and rotatable bonds in later quinolones, whereas logP, TPSA, and Csp^3^ did not differ significantly across generations.

## 9. Concluding Remarks

The literature attributes the discovery of NA to chance by Lesher and colleagues in the 1960s, in which this drug was identified as a by-product in the synthesis of chloroquine. Nonetheless, the history of the quinolone scaffold may be traced back to the alkaloids isolated from *Medicosma fareana* by Crow and Price in the late 1940s. Since then, several others were proposed, synthesized and launched on the market over a period of more than sixty years, encompassing four different generations. This remarkable class expanded the range of antibacterial drugs available beyond β-lactams and sulfonamides, allowing the treatment of many Gram-negative bacterial infections.

The subject of quinolones is vast, as this pharmacophore is highly attractive to medicinal chemists. Various research groups are exploring new modifications at permissible positions (cf. the SAR in Figure 16), consistently aiming to broaden the spectrum of action, enhance potency, and optimize dosing regimens. Other groups are investigating different biological activities and therapeutic targets—for instance, with the goal of developing novel antitumor agents. All these efforts require new synthetic routes, analytical methods, and pharmacological, pharmacokinetic, and toxicological evaluations. This emphasizes the significance of quinolones and warrants a separate manuscript dedicated exclusively to their future prospects.

Throughout this review, we highlighted several important aspects related to the chemical development and historical background of quinolones, including a bibliometric analysis of scientific production from 1945 to 2025. Moreover, we discussed the pharmacodynamics and SAR aspects of quinolones, encompassing their mechanisms of actions in DNA Gyrase and Topo IV, illustrated through molecular docking simulations of twenty quinolones and their interactions within the binding sites. Furthermore, we explored the pharmacokinetic features of each generation, emphasizing the most important drugs and discussing the pharmacokinetic/pharmacodynamics (PK/PD) index, which demonstrates that quinolones act as concentration-dependent antimicrobials. We also included toxicological aspects of this drug class, focusing on the most important adverse effects and the drugs that have been withdrawn from the market. Through this review we aim to guide the readers, especially students of medicinal chemistry and pharmacology, through the development, mechanisms of action and pharmacology of quinolones, thereby complementing their knowledge.

## Figures and Tables

**Table 1:** Current status of marketed quinolones for human use in different countries.

Quinolones	BRAZIL (ANVISA)	USA (FDA)	FRANCE (ANSM)	GERMANY (BfARM)	JAPAN (PMDA)	UK (MHRA)
Nalidixic acid (NA)	Active	Discontinued	Discontinued	Discontinued	NRF	NRF
Ciprofloxacin (CIP)	Active	Active	Active	Active	Active	Active
Norfloxacin (NOR)	Active	Discontinued	Active	Active	NRF	NRF
Ofloxacin (OFLX)	Active	Active	Active	Active	NRF	Active
Levofloxacin (LEV)	Active	Active	Active	Active	Active	Active
Pazufloxacin (PZFX)	NRF	NRF	NRF	NRF	Active	NRF
Tosufloxacin (TFLX)	NRF	NRF	NRF	NRF	Active	NRF
Besifloxacin (BSF)	NRF	Active (ocular)	NRF	NRF	NRF	NRF

NRF: No record found; FDA: Food and Drug Administration (USA); ANVISA: Agência Nacional de Vigilância Sanitária (Brazil); Agence Nationale de Securité du Médicament et des Produits de Santé (France); MHRA: Medicines & Healthcare Products Regulatory Agency (UK); BfARM: Bundesinstitut für Arzneimittel und Medizinprodukte (Germany); PMDA: Pharmaceuticals and Medical Devices Agency (Japan).

**Table 2:** Summary of *in vitro* tests performed with OFLX and its two enantiomers *S*-(-)-ofloxacin and *R*-(+)-ofloxacin against ten bacterial strains.

Strains	Ofloxacin (Racemate)	*S*-Ofloxacin DR3355 (LEV)	*R*-Ofloxacin DR3354	*S* Is More Active Than *R*	*S* Is More Active Than Racemate
	MIC (µg/mL)				
*Enterobacter cloacae*	≤0.05	≤0.05	0.78	(15.6x)	Similar
*Escherichia coli*	≤0.05	≤0.05	0.39	(7.8x)	Similar
*Klebsiella pneumoniae*	0.10	≤0.05	1.56	(31.2x)	2x
*Proteus vulgaris*	≤0.05	≤0.05	0.39	(7.8x)	Similar
*Pseudomonas aeruginosa*	0.20	0.10	6.25	(62.5x)	2x
*Serratia marcescens*	0.10	≤0.05	1.56	(31.2x)	2x
*Staphylococcus aureus*	0.39	0.20	25.0	(125x)	1.95x
*Staphylococcus epidermidis*	0.39	0.39	25.0	(64.1x)	Similar
*Streptococcus faecalis*	3.13	1.56	>100	-	2x
*Streptococcus pyogenes*	3.13	1.56	>100	-	2x

## Data Availability

The data supporting the findings of this review are included in the article and its Supplementary Materials. Additional data may be obtained from the corresponding author upon reasonable request.

## Supplementary Materials

The following supporting information can be downloaded at: https://www.mdpi.com/article/10.3390/molecules31213949/s1, Figure S1: Molecular docking of quinolones within the *E. coli* DNA gyrase–DNA complex (PDB ID: 9GGQ), showing (A) the binding pocket containing the co-crystallized ligand moxifloxacin and (B) the superimposition of the docked quinolones; Figure S2: Molecular docking of quinolones within the *S. pneumoniae* topoisomerase IV–DNA complex (PDB ID: 8QMB), showing (A) the binding pocket containing the co-crystallized ligand delafloxacin and (B) the superimposition of the docked quinolones; Table S1: Molecular docking results, redocking validation, and protein–ligand interaction profiles of quinolones with DNA gyrase and topoisomerase IV; Table S2: Inhibitory activity (IC_50_) of quinolones against bacterial DNA gyrase and topoisomerase IV; Table S3: Antibacterial spectrum and therapeutic indications of quinolones; Table S4: Pharmacokinetic parameters of quinolones used in systemic antimicrobial therapy; Table S5: Tissue penetration and pharmacokinetic/pharmacodynamic (PK/PD) indices of selected quinolones; Table S6: Physicochemical descriptors of quinolones used in the drug-like property analysis; Supplementary File S1: Search strategy for the bibliometric analysis of quinolone antimicrobials.

## Abbreviations

The following abbreviations are used in this manuscript:

**Abbreviation**	**Definition**
ABC	ATP-binding cassette
ANSM	Agence Nationale de Sécurité du Médicament et des Produits de Santé
ANVISA	Agência Nacional de Vigilância Sanitária
ATP	Adenosine triphosphate
AUC	Area under the concentration–time curve
BfArM	Bundesinstitut für Arzneimittel und Medizinprodukte
BPAA	Biphenylacetic acid
BSF	Besifloxacin
CIP	Ciprofloxacin
CNS	Central nervous system
Csp^3^	Fraction of sp^3^-hybridized carbons
CYP	Cytochrome P450
CYP1A2	Cytochrome P450 1A2
CYP2C8	Cytochrome P450 2C8
CYP2C9	Cytochrome P450 2C9
CYP3A4	Cytochrome P450 3A4
EMA	European Medicines Agency
ENX	Enoxacin
FDA	Food and Drug Administration
FLU	Flumequine
FQ	Fluoroquinolone
GABA	γ-Aminobutyric acid
GAT	Gatifloxacin
GLASS	Global Antimicrobial Resistance and Use Surveillance System
GMFX	Gemifloxacin
HBA	Hydrogen-bond acceptor
HBD	Hydrogen-bond donor
hERG	Human ether-à-go-go-related gene
HPLC	High-performance liquid chromatography
IC_50_	Half-maximal inhibitory concentration
LEV	Levofloxacin
LSFX	Lascufloxacin
MATE	Multidrug and toxic compound extrusion
MDR	Multidrug resistance
MDR-TB	Multidrug-resistant tuberculosis
MFS	Major facilitator superfamily
MHRA	Medicines and Healthcare products Regulatory Agency
MIC	Minimum inhibitory concentration
MXF	Moxifloxacin
MW	Molecular weight
NA	Nalidixic acid
NMDA	N-Methyl-D-aspartate
NMR	Nuclear magnetic resonance
NOR	Norfloxacin
NRF	No record found
OFLX	Ofloxacin
PCA	Principal component analysis
PDB	Protein Data Bank
PDBID	Protein Data Bank identifier
PD	Pharmacodynamics
PFL	Pefloxacin
PK	Pharmacokinetics
PK/PD	Pharmacokinetics/pharmacodynamics
PMDA	Pharmaceuticals and Medical Devices Agency
PPA	Pipemidic acid
PRu	Unbound tissue-to-plasma penetration ratio
PZFX	Pazufloxacin
QRDR	Quinolone resistance-determining region
RB	Rotatable bonds
RFX	Rufloxacin
RMSD	Root-mean-square deviation
RND	Resistance-nodulation-division
ROS	Reactive oxygen species
SAR	Structure–activity relationship
SPFX	Sparfloxacin
STFX	Sitafloxacin
TFLX	Tosufloxacin
TMFX	Temafloxacin
TMQR	Transferable mechanisms of quinolone resistance
Topo IV	Topoisomerase IV
TOPRIM	Topoisomerase-primase domain
TPSA	Topological polar surface area
TRO	Trovafloxacin
UVA	Ultraviolet A
WHO	World Health Organization
