# Supplementary material for: Quinolone Antimicrobials: A Medicinal Chemistry Perspective on Structural Evolution, Pharmacology, and Computational Profiling
Source: Molecules. 2026 Nov 2;31(21):3949. doi: 10.3390/molecules31213949 (PMC13765324; doi:10.3390/molecules31213949)
Supplement: Supplementary file 1 [file molecules-31-03949-s001.zip › Supplementary File S1 search_strategy.pdf]

molecules

Review

# Quinolone Antimicrobials: A Medicinal Chemistry Perspective on Structural Evolution, Pharmacology, and Computational Profiling

**Gustavo Machado das Neves [1,2,†], Graziela de Araújo Lock [3,†], Itamar Luís Gonçalves [4,†], Teresa Cristina Tavares Dalla Costa [5] and Vera Lucia Eifler-Lima [1,*]**

1 Medicinal Organic Synthesis Laboratory (LaSOM®), Pharmaceutical Sciences Graduate Program, Faculty of Pharmacy, Federal University of Rio Grande do Sul, Porto Alegre 90610-000, Brazil; gus.mneves@gmail.com
2 Faculty of Medicine, Federal University of Rio Grande, Rio Grande 96200-200, Brazil
3 Department of Pharmacy, Health Sciences Centre, Federal University of Santa Maria, Santa Maria 97105-900, Brazil; graziela.lock@gmail.com
4 Graduate Program in Integral Health Care (UNICRUZ/URI-Erechim/UNIJUÍ), Integrated Regional University of Alto Uruguai e das Missões, Erechim 99709-910, Brazil; itamar3141@yahoo.com.br
5 Pharmacokinetics and PK/PD Modeling Laboratory, Faculty of Pharmacy, Federal University of Rio Grande do Sul, Porto Alegre 90610-000, Brazil; dalla.costa@ufrgs.br
* Correspondence: veraeifler@ufrgs.br
† These authors contributed equally to this work.

## Search Strategy for the Bibliometric Analysis of Quinolone Antimicrobials

("Nalidixic acid" OR "Negram" OR "Urisal" OR "Nalidix" OR "Nalidixin" OR "389-08-2" OR "1-ethyl-7-methyl-4-oxo-1,8-naphthyridine-3-carboxylic acid") OR

("Ciprofloxacin" OR "Cipro" OR "Ciproxin" OR "Ciprobay" OR "Proquin XR" OR "Ciflox" OR "Ciprinol" OR "85721-33-1" OR "1-cyclopropyl-6-fluoro-4-oxo-7-(piperazin-1-yl)-1,4-dihydroquinoline-3-carboxylic acid") OR

("Norfloxacin" OR "Noroxin" OR "Chibroxin" OR "Lexinor" OR "Norflox" OR "70458-96-7" OR "1-ethyl-6-fluoro-4-oxo-7-piperazin-1-yl-1,4-dihydroquinoline-3-carboxylic acid") OR

("Ofloxacin" OR "Floxin" OR "Ocuflox" OR "Tarivid" OR "Zanocin" OR "Oflocet" OR "82419-36-1" OR "(RS)-7-fluoro-2-methyl-6-(4-methylpiperazin-1-yl)-10-oxo-4-oxa-1-azatricyclo[7.3.1.0^5,13]trideca-5(13),6,8,11-tetraene-11-carboxylic acid") OR

("Levofloxacin" OR "Levaquin" OR "Tavanic" OR "Cravit" OR "Iquix" OR "100986-85-4" OR "(S)-9-fluoro-2,3-dihydro-3-methyl-10-(4-methylpiperazin-1-yl)-7-oxo-7H-pyrido[1,2,3-de]-1,4-benzoxazine-6-carboxylic acid") OR

("Besifloxacin" OR "Besivance" OR "141388-60-1" OR "7-[(3R,4S)-3-amino-4-methylpyrrolidin-1-yl]-1-cyclopropyl-6-fluoro-8-chloro-1,4-dihydro-4-oxoquinoline-3-carboxylic acid") OR

("Gatifloxacin" OR "Gatiflo" OR "Tequin" OR "Zymar" OR "112811-59-3" OR "1-cyclopropyl-6-fluoro-8-methoxy-7-(3-methylpiperazin-1-yl)-4-oxoquinoline-3-carboxylic acid") OR

("Moxifloxacin" OR "Avelox" OR "Vigamox" OR "Moxiflox" OR "151096-09-2" OR "1-cyclopropyl-7-[(1S,6S)-2,8-diazabicyclo[4.3.0]nonan-8-yl]-6-fluoro-8-methoxy-4-oxoquinoline-3-carboxylic acid") OR

("Pazufloxacin" OR "Pasil" OR "Pazucross" OR "127045-41-4" OR "(3R)-10-(1-aminocyclopropyl)-9-fluoro-3-methyl-7-oxo-1H,7H-[1,3]oxazino[5,4,3-ij]quinoline-carboxylic acid") OR

("Tosufloxacin" OR "Ozex" OR "Tosuxacin" OR "100490-36-6" OR "7-(3-amino-1-pyrrolidinyl)-1-(2,4-difluorophenyl)-6-fluoro-4-oxo-1,4-dihydro-1,8-naphthyridine-3-carboxylic acid") OR

("Lascufloxacin" OR "Lasvic" OR "848416-07-9" OR "7-[(3S,4S)-3-[(cyclopropylamino)methyl]-4-fluoropyrrolidin-1-yl]-6-fluoro-1-(2-fluoroethyl)-8-methoxy-4-oxoquinoline-3-carboxylic acid") OR

("Sitafloxacin" OR "Gracevit" OR "127254-12-0" OR "7-[(7S)-7-amino-5-azaspiro[2.4]heptan-5-yl]-8-chloro-6-fluoro-1-[(1R,2S)-2-fluorocyclopropyl]-4-oxoquinoline-3-carboxylic acid")
