# Supplementary material for: Quinolone Antimicrobials: A Medicinal Chemistry Perspective on Structural Evolution, Pharmacology, and Computational Profiling
Source: Molecules. 2026 Nov 2;31(21):3949. doi: 10.3390/molecules31213949 (PMC13765324; doi:10.3390/molecules31213949)
Supplement: Supplementary file 1 [file molecules-31-03949-s001.zip › Supplementary Table S3.pdf]

Supplementary Table S3: Quinolone´s spectrum of activity and therapeutic indications.

Quinolone	Activity against Gram-negative	Activity against Gram-positive	Indication	Ref.
**First generation**				
Nalidixic acid	*Enterobacteriaceae (Escherichia coli, Klebsiella spp., Proteus spp.).*	Insignificant.	Uncomplicated lower urinary tract infections.	[1, 2]
**Second generation**				
Ciprofloxacin	*Pseudo*monas aeruginosa, Enterobacteriaceae (*E. coli* - including many resistant strains), *K. pneumoniae, Enterobacter, Salmonella, Shigella, Citrobacter, Haemophilus influenza,* Legionella, *Neisseria gonorrhoeae*, *Campylobacter, Proteus mirabilis, Moraxella catarrhalis.*	*Staphylococcus aureus* (including some methicillin-susceptible strains), *Streptococcus pneumoniae.*	Urinary tract infections, sexually transmitted infections (gonorrhea and chancroid), skin and soft tissue infection, bone and joint infections, prostatitis, pneumonia, typhoid fever, gastrointestinal infections, lower respiratory tract infections, inhalation anthrax (post-exposure prophylaxis), plague, and salmonellosis, acute bacterial exacerbation of chronic bronchitis.	[3-12]
Ofloxacin	*Enterobacteriaceae* (*E. coli, K. pneumoniae, Serratia marcescens*, *Providencia* spp.), *P. mirabilis H. influenzae, N. gonorrhoeae, C. trachomatis.*	*S. aureus* (including methicillin-resistant strains); *Streptococci* and *Enterococci* (limited for both).	Acute bacterial exacerbations of chronic bronchitis, community-acquired pneumonia, uncomplicated skin and skin structure infections, acute uncomplicated urethral/cervical gonorrhea and nongonococcal urethritis/cervicitis, acute pelvic inflammatory disease.	[13-15]

			Topic usage for: bacterial conjunctivitis and corneal ulcers caused by susceptible strains of gram-positive and gram-negative bacteria, otitis externa, chronic suppurative otitis media, acute otitis media.	
Norfloxacin	E*nterobacteriaceae (E. coli, K. pneumoniae) P. mirabilis*, *P. aeruginosa*, Enterobacter spp. *N. gonorrhoeae.*	*S. aureus* (including some resistant strains).	Uncomplicated and complicated urinary tract infections such as cystitis, pyelonephritis, prostatitis, bacterial gastroenteritis and traveller's diarrhea, gonorrhea.	[16-20]
**Third generation**				
Levofloxacin	*Enterobacteriaceae (E. coli, Klebsiella* spp., *Proteus spp.*), *Haemophilus influenzae*, *M. catarrhalis*, *P. aeruginosa*.	*S. pneumoniae* (including penicillin-resistant and macrolide-resistant strains), methicillin-susceptible *S. aureus* (MSSA), *Enterococcus* spp.	Respiratory tract infections (community-acquired pneumonia - including multi-drug resistant), nosocomial (hospital-acquired) pneumonia, acute bacterial exacerbation of chronic bronchitis, and acute sinusitis, urinary tract infections complicated and uncomplicated, as well as acute pyelonephritis, complicated and uncomplicated skin and skin structure infections (abscesses, cellulitis),	[3, 21-25]

			chronic bacterial prostatitis, plague (including pneumonic and septicemic) and prophylaxis/treatment of inhalational anthrax.	
**Fourth generation**				
Moxifloxacin**	*H. influenzae, M. catarrhalis, E. coli, K. pneumoniae, P. mirabilis, Bacteroides* spp., Clostridium* spp., C. pneumoniae.*	*S. pneumoniae* (including penicillin/macrolide-resistant), *S. aureus* (methicillin-susceptible only), *Streptococcus pyogenes, Mycobacterium* spp.	Respiratory infections like community-acquired pneumonia (including multi-drug-resistant strains), acute bacterial sinusitis, and acute exacerbations of chronic bronchitis, uncomplicated and complicated skin and skin structure infections (cellulitis, abscesses), complicated intra-abdominal infections, including polymicrobial abscesses, plague (pneumonic and septicemic), mild to moderate pelvic inflammatory disease (without tubo-ovarian abscess).	[26-33]
Gemifloxacin	*H. influenzae, M. catarrhalis, K. pneumoniae, E. coli, Enterobacter cloacae*, *Proteus spp.*, *Chlamydia pneumoniae*	*Streptococcus pneumoniae* (including penicillin-resistant strains), *Staphylococcus aureus* (methicillin-susceptible), *Streptococcus pyogenes*, *Streptococcus viridans*, *Legionella pneumophila*	Acute bacterial exacerbation of chronic bronchitis and mild-to-moderate community-acquired pneumonia. It is generally reserved for patients with no alternative treatment options due to	[28, 34-37]

			potential, serious side effects.	
Pazufloxacin	*E. coli, H influenzae, K. pneumoniae, M. catharralis, P. mirabilis, Serratia marcescens*, Citrobacter sp., Shigella sp., Salmonella sp., Aeromonas sp., *P. aeruginosa, Aeromonas sp. and Yersinia.*	*S. aureus* (including MRSA), *S. epidermidis* (including MRSE), *S. pneumoniae*.	Respiratory tract infections like pneumonia and chronic respiratory disease infections, urinary tract infections like complicated or severe pyelonephritis and cystitis, peritonitis, cholecystitis, ophthalmic preparations for conjunctivitis	[38-44]
Tosufloxacin	*H. influenza, Salmonella spp., Shigella spp., Campylobacter spp., Vibrio spp., P. aeruginosa*	*S. aureus, S. epidermidis, Streptococci (including penicillin-resistant S. pneumoniae (PRSP) and intermediate strains (PISP), Enterococcus*	Respiratory infections such as pneumonia, chronic bronchitis, pharyngitis/laryngitis, and tonsillitis; urinary tract infections as pyelonephritis, prostatitis, urethritis, and bartholinitis, deep-seated skin infections, acne, lymphangitis, mastitis, and abscesses; otitis media and sinusitis, cholera, salmonella,	[45-52]

			and sometimes for anthrax; otitis media and other infections in children.	
Lascufloxacin*	*H influenzae, M. catarrhalis, Bacteroides fragilis**	*S. aureus* (including MRSA), *S. epidermidis*, *S. pneumoniae* (including penicillin-resistant strains), *Clostridium difficile*, Clostridium perfringens**	Community-acquired pneumonia, acute exacerbation of chronic bronchitis, and various ear, nose and throat infections (sinusitis, otitis media, tonsillitis, pharyngolaryngitis).	[53, 54]
Sitafloxacin	*Enterobacteriaceae* (*E. coli*, Klebsiella spp., *Enterobacter* spp.), *H. influenzae*, *M. catarrhalis*, *P. aeruginosa, B. fragilis*, Prevotella spp., Fusobacterium** spp., *Mycoplasma pneumoniae*, *C. trachomatis*	*S. pneumoniae* (including resistant strains), *S. aureus* (MSSA), and *Enterococcus faecalis*.	Respiratory tract infections such as community-acquired pneumonia, acute bronchitis, chronic respiratory disease, and sinusitis, complicated and uncomplicated urinary tract infections (cystitis, pyelonephritis), prostatitis, and cervicitis/urethritis, pericoronitis, periodontitis, and inflammation of the jaw (osteitis of the jaw), otorhinological infections: otitis media.	[55-59]

Gatifloxacin**	*H. influenzae, M. catarrhalis*, *Neisseria* spp., *Enterobacteriaceae* (*E. coli*), *B. fragilis*, Legionella spp., Chlamydia spp*	*S. pneumoniae* (including PRSP), *S. aureus* (MSSA), *S. epidermidis*.	Bacterial conjunctivitis<br>For the treatment of bronchitis, sinusitis, community-acquired pneumonia, and skin infections (abscesses, wounds)	[33, 60-63]
Besifloxacin	*H. influenzae, M. catarrhalis, P. aeruginosa*.	*S. aureus* (including MRSA), methicillin-resistant *S. epidermidis*, *Staphylococcus hominis, S. pneumoniae, Streptococcus oralis. Corynebacterium spp. (C. striatum, C. pseudodiphtheriticum)*	Bacterial conjunctivitis	[64-67]

**Active against *Mycoplasma pneumoniae*

Anaerobic bacteria

References

[1] G.C. Crumplin, J.T. Smith, Nalidixic acid: an antibacterial paradox, Antimicrobial Agents and Chemotherapy, 8 (1975) 251-261.

[2] DrugBank, Nalidixic acid [DB00779], (2026).

[3] P.G. Ambrose, S.M. Bhavnani, R.C. Owens, Jr., Clinical pharmacodynamics of quinolones, Infectious Disease Clinics of North America, 17 (2003) 529-543.

[4] A. Meyerhoff, R. Albrecht, J.M. Meyer, P. Dionne, K. Higgins, D. Murphy, US Food and Drug Administration approval of ciprofloxacin hydrochloride for management of postexposure inhalational anthrax, Clinical Infectious Diseases, 39 (2004) 303-308.

[5] T. Apangu, K. Griffith, J. Abaru, G. Candini, H. Apio, F. Okoth, R. Okello, J. Kaggwa, S. Acayo, G. Ezama, B. Yockey, C. Sexton, M. Schriefer, E.K. Mbidde, P. Mead, Successful Treatment of Human Plague with Oral Ciprofloxacin, Emerging Infectious Diseases, 23 (2017) 553-555.

[6] M. Unemo, M.M. Lahra, M. Cole, P. Galarza, F. Ndowa, I. Martin, J.R. Dillon, P. Ramon-Pardo, G. Bolan, T. Wi, World Health Organization Global Gonococcal Antimicrobial Surveillance Program (WHO GASP): review of new data and evidence to inform international collaborative actions and research efforts, Sexual Health, 16 (2019) 412-425.

[7] DrugBank, Ciprofloxacin [DB00537], (2026).

[8] T. Thai, B.H. Salisbury, P.M. Zito, Ciprofloxacin, in: StatPearls [Internet], StatPearls Publishing, Treasure Island (FL), 2026.

[9] A.A. Lopes, K. Salgado, R. Martinelli, H. Rocha, Aumento da freqüência de resistência à norfloxacina e ciprofloxacina em bactérias isoladas em uroculturas, Revista da Associação Médica Brasileira, 44 (1998) 196-200.

[10] J. Kwiecinska-Pirog, K. Skowron, W. Bartczak, E. Gospodarek-Komkowska, The Ciprofloxacin Impact on Biofilm Formation by Proteus Mirabilis and P. Vulgaris Strains, Jundishapur Journal of Microbiology, 9 (2016) e32656.

[11] W.H. Traub, B. Leonhard, Susceptibility of Moraxella catarrhalis to 21 antimicrobial drugs: validity of current NCCLS criteria for the interpretation of agar disk diffusion antibiograms, Chemotherapy, 43 (1997) 159-167.

[12] L. Zabel, W.D. Hirschmann, Ciprofloxacin in der Behandlung der Legionärskrankheit [Ciprofloxacin in the treatment of Legionnaires' disease], Pneumologie, 44 (1990) 826-828.

[13] E.J. Perea, Ofloxacin concentrations in tissues involved in respiratory tract infections, Journal of Antimicrobial Chemotherapy, 26 Suppl D (1990) 55-60.

[14] DrugBank, Ofloxacin [DB01165], (2026).

[15] D.B. Graham, J. Tripp, Ofloxacin, in: StatPearls [Internet], StatPearls Publishing, Treasure Island (FL), 2026.

[16] R.C. Rowen, D.J. Michel, J.C. Thompson, Norfloxacin: clinical pharmacology and clinical use, Pharmacotherapy, 7 (1987) 92-110.

[17] DrugBank, Norfloxacin [DB01059], (2026).

[18] J. Joost, Norfloxacin in der Behandlung von Harnwegsinfekten [Norfloxacin in the treatment of urinary tract infections], Wiener Klinische Wochenschrift, 98 (1986) 355-357.

[19] S.R. Norrby, M. Jonsson, Antibacterial activity of norfloxacin, Antimicrobial Agents and Chemotherapy, 23 (1983) 15-18.

[20] C. Lee, A.R. Ronald, Norfloxacin: its potential in clinical practice, The American Journal of Medicine, 82 (1987) 27-34.

[21] DrugBank, Levofloxacin [DB01137], (2026).

[22] M. García-Solache, L.B. Rice, The Enterococcus: a Model of Adaptability to Its Environment, Clinical Microbiology Reviews, 32 (2019).

[23] J.C. McGregor, G.P. Allen, D.T. Bearden, Levofloxacin in the treatment of complicated urinary tract infections and acute pyelonephritis, Therapeutics and Clinical Risk Management, 4 (2008) 843-853.

[24] O. Murillo, M.E. Pachón, G. Euba, R. Verdaguer, F. Tubau, C. Cabellos, J. Cabo, F. Gudiol, J. Ariza, High doses of levofloxacin vs moxifloxacin against staphylococcal experimental foreign-body infection: the effect of higher MIC-related pharmacokinetic parameters on efficacy, Journal of Infection, 58 (2009) 220-226.

[25] V. Podder, P. Patel, N.M. Sadiq, Levofloxacin, in: StatPearls [Internet], StatPearls Publishing, Treasure Island (FL), 2026.

[26] K. Betlejewska, W. Hryniewicz, Susceptibility to moxifloxacin and other antimicrobial agents of major pathogens responsible for community acquired respiratory tract infection in Poland, International Journal of Antimicrobial Agents, 19 (2002) 47-52.

[27] J.R. Bogner, A. Kutaiman, M. Esguerra-Alcalen, S. Heldner, P. Arvis, Moxifloxacin in complicated skin and skin structure infections (cSSSIs): A prospective, international, non-interventional, observational study, Advances in Therapy, 30 (2013) 630-643.

[28] C.M. Culley, M.K. Lacy, N. Klutman, B. Edwards, Moxifloxacin: clinical efficacy and safety, American Journal of Health-System Pharmacy, 58 (2001) 379-388.

[29] DrugBank, Moxifloxacin [DB00218], (2026).

[30] Z.E. Humma, P. Patel, Moxifloxacin, in: StatPearls [Internet], StatPearls Publishing, Treasure Island (FL), 2026.

[31] R.B. Muijsers, B. Jarvis, Moxifloxacin in uncomplicated skin and skin structure infections, Drugs, 62 (2002) 967-973.

[32] M. Müller, H. Stass, M. Brunner, J.G. Möller, E. Lackner, H.G. Eichler, Penetration of moxifloxacin into peripheral compartments in humans, Antimicrobial Agents and Chemotherapy, 43 (1999) 2345-2349.

[33] G. Torkildsen, J.W. Proksch, A. Shapiro, S.K. Lynch, T.L. Comstock, Concentrations of besifloxacin, gatifloxacin, and moxifloxacin in human conjunctiva after topical ocular administration, Clinical Ophthalmology, 4 (2010) 331-341.

[34] J.M. Blondeau, B. Missaghi, Gemifloxacin: a new fluoroquinolone, Expert Opinion on Pharmacotherapy, 5 (2004) 1117-1152.

[35] DrugBank, Gemifloxacin [DB01155], (2026).

[36] D.J. Hoban, S.K. Bouchillon, J.L. Johnson, G.G. Zhanel, D.L. Butler, L.A. Miller, J.A. Poupard, Comparative in vitro activity of gemifloxacin, ciprofloxacin, levofloxacin and ofloxacin in a North American surveillance study, Diagnostic Microbiology and Infectious Disease, 40 (2001) 51-57.

[37] R.A. Press, The use of fluoroquinolones as antiinfective transition-therapy agents in community-acquired pneumonia, Pharmacotherapy, 21 (2001) 100S-104S.

[38] L. Baiza-Durán, O. Olvera-Montaño, A.R. Mercado-Sesma, A.A. Oregon-Miranda, A. Lizárraga-Corona, J.C. Ochoa-Tabares, A.L. Pérez-Balbuena, I.M. Montoya-Sánchez, L.R. Saucedo-Rodríguez, A. Mora-González, P.A. Gómez-Bastar, M.A. Villanueva-Najera, L.I. Sandoval-Delgadillo, M. González-Lomelí, J.H. Páez-Garza, M. Orozco-Carroll, M. Casillas-Magallanes, Efficacy and Safety of 0.6% Pazufloxacin Ophthalmic Solution Versus Moxifloxacin 0.5% and Gatifloxacin 0.5% in Subjects with Bacterial Conjunctivitis: A Randomized Clinical Trial, Journal of Ocular Pharmacology and Therapeutics, 34 (2018) 250-255.

[39] DrugBank, Pazufloxacin [DB11774], (2026).

[40] Y. Kodama, S. Hori, S. Tominaga, A. Ohwada, K. Yoshimi, M. Sekiya, M. Yamazaki, T. Mori, T. Suzuki, Y. Fukuchi, K. Takahashi, Evaluation of clinical effectiveness of Pazufloxacin Mesilate in acute exacerbation of chronic respiratory diseases, Nihon Kokyuki Gakkai Zasshi, 46 (2008) 781-787.

[41] S. Kohno, N. Aoki, S. Kawai, Y. Niki, A. Watanabe, S. Hori, S. Watanabe, K. Totsuka, A clinical phase III study of pazufloxacin in patients with bacterial pneumonia, Japanese Journal of Chemotherapy, 58 (2010).

[42] J. Mitsuyama, H. Yamada, J. Maehana, Y. Fukuda, S. Kurose, S. Minami, Y. Todo, Y. Watanabe, H. Narita, Characteristics of quinolone-induced small colony variants in Staphylococcus aureus, Journal of Antimicrobial Chemotherapy, 39 (1997) 697-705.

[43] Y. Umezaki, K. Matsumoto, K. Ikawa, Y. Yokoyama, Y. Enoki, A. Shigemi, E. Watanabe, K. Nakamura, K. Ueno, H. Terazono, N. Morikawa, Y. Takeda, Concentration-Dependent Activity of Pazufloxacin against Pseudomonas aeruginosa: An In Vivo Pharmacokinetic/Pharmacodynamic Study, Antibiotics, 11 (2022) 982.

[44] NIH, NCATS/NIH, I. Division of Preclinical, Pazufloxacin [4CZ1R38NDI], (2026).

[45] J.P. Bryan, C. Waters, J. Sheffield, R.E. Krieg, P.L. Perine, K. Wagner, In vitro activities of tosufloxacin, temafloxacin, and A-56620 against pathogens of diarrhea, Antimicrobial Agents and Chemotherapy, 34 (1990) 368-370.

[46] I. Kobayashi, K. Matsuzaki, K. Omika, M. Hasegawa, Y. Sato, Antibacterial activity of tosufloxacin against major organisms detected from patients with respiratory infections, Japanese Journal of Antibiotics, 60 (2007) 98-106.

[47] R. Minami, N. Inotsume, C. Nakamura, M. Nakano, Stereoselective analysis of the disposition of tosufloxacin enantiomers in man, European Journal of Clinical Pharmacology, 45 (1993) 489-491.

[48] Y. Niki, Pharmacokinetics and safety assessment of tosufloxacin tosilate, Journal of Infection and Chemotherapy, 8 (2002) 1-18.

[49] Y. Onodera, J. Okuda, M. Tanaka, K. Sato, Inhibitory activities of quinolones against DNA gyrase and topoisomerase IV of Enterococcus faecalis, Antimicrobial Agents and Chemotherapy, 46 (2002) 1800-1804.

[50] S. Yokota, Y. Ohkoshi, K. Sato, N. Fujii, Emergence of fluoroquinolone-resistant Haemophilus influenzae strains among elderly patients but not among children, Journal of Clinical Microbiology, 46 (2008) 361-365.

[51] S. Yong, M. Yang, M. Zhou, Efficacy and safety review of tosufloxacin in the treatment of bacterial infection in children, European Journal of Clinical Microbiology & Infectious Diseases, (2025).

[52] Y. Yamashiro, M. Nunome, M. Takahata, T. Yasuda, Penetration into bone and joint tissues of rabbits and in vitro antibacterial activity of tosufloxacin, The Japanese Journal of Antibiotics, 46 (1993) 31-35.

[53] J. Hirai, Successful Outpatient Treatment of Legionella pneumophila Pneumonia With Lascufloxacin: A Case Report, Cureus, 17 (2025) e81207.

[54] K. Tanaka, H. Vu, M. Hayashi, In vitro activities and spectrum of lascufloxacin (KRP-AM1977) against anaerobes, Journal of Infection and Chemotherapy, 27 (2021) 1265-1269.

[55] DrugBank, Sitafloxacin [DB13261], (2026).

[56] S. Kamidoro, T. Matsui, K. Tanaka, S. Arakawa, M. Fujisawa, Study on the penetration of sitafloxacin into the prostatic fluid, Japanese Journal of Chemotherapy, 60 (2012).

[57] G.M. Keating, Sitafloxacin: in bacterial infections, Drugs, 71 (2011) 731-744.

[58] E.M.A. Kuhn, L.A. Sominsky, M. Chittò, E.M. Schwarz, T.F. Moriarty, Antibacterial Mechanisms and Clinical Impact of Sitafloxacin, Pharmaceuticals, 17 (2024) 1537.

[59] S. Matsuda, M. Noguchi, J. Yasuda, S. Hori, Clinical Study of sitafloxacin in treatment of cervicitis with Chlamydia tracomatis, Japanese Journal of Chemotherapy, 56 (2008).

[60] DrugBank, Gatifloxacin [DB01044], (2026).

[61] D.M. Grasela, Clinical pharmacology of gatifloxacin, a new fluoroquinolone, Clinical Infectious Diseases, 31 Suppl 2 (2000) S51-S58.

[62] E. Huczko, B. Conetta, D. Bonner, L. Valera, T. Stickle, A. Macko, J. Fung-Tomc, Susceptibility of bacterial isolates to gatifloxacin and ciprofloxacin from clinical trials 1997-1998, International Journal of Antimicrobial Agents, 16 (2000) 401-405.

[63] C. Schultz, Gatifloxacin ophthalmic solution for treatment of bacterial conjunctivitis: safety, efficacy and patient perspective, Ophthalmology and Eye Diseases, 4 (2012) 65-70.

[64] M.H. Chang, H.B. Fung, Besifloxacin: a topical fluoroquinolone for the treatment of bacterial conjunctivitis, Clinical Therapeutics, 32 (2010) 454-471.

[65] DrugBank, Besifloxacin [DB06771], (2026).

[66] C.M. Sanfilippo, C.M. Allaire, H.H. DeCory, Besifloxacin Ophthalmic Suspension 0.6% Compared with Gatifloxacin Ophthalmic Solution 0.3% for the Treatment of Bacterial Conjunctivitis in Neonates, Drugs in R&D, 17 (2017) 167-175.

[67] B.E. Silverstein, T.W. Morris, L.S. Gearinger, H.H. Decory, T.L. Comstock, Besifloxacin ophthalmic suspension 0.6% in the treatment of bacterial conjunctivitis patients with Pseudomonas aeruginosa infections, Clinical Ophthalmology, 6 (2012) 1987-1996.
