# Supplementary material for: Quinolone Antimicrobials: A Medicinal Chemistry Perspective on Structural Evolution, Pharmacology, and Computational Profiling
Source: Molecules. 2026 Nov 2;31(21):3949. doi: 10.3390/molecules31213949 (PMC13765324; doi:10.3390/molecules31213949)
Supplement: Supplementary file 1 [file molecules-31-03949-s001.zip › Supplementary Table S4.pdf]

Supplementary Table S4. Pharmacokinetics of quinolones used systemically in human´s antimicrobial therapy.

Quinolones	Dose/via	Protein binding (%)	$F_{abs,oral}$	Vd (L/kg)	CL (L/h)	Elimination route	$t_{1/2}$	Substrate to transporters	References
**First generation**									
Nalidixic Acid (NA)	1000 mg p.o.	90-97	96	1	2.9 ± 0.6 (older subjects) ***<br>8.3 ± 1.8 (young subjects) ***	Metabolism (42% glucuronidated, 40% hydroxylated); Renal (2-3%)	2.7 ± 0.7 h (young subjects) **<br>6-7h ****	OAT1 and OAT3**	[1-6]
**Second generation**									
Ciprofloxacin (CIP)	750 mg p.o.	15-25	70-80	2.0-3.5	36.0 – 42.6*	Renal (40-60%); metabolism (15%), faeces (15-40%)	5.27 ± 1.14	OATP1A2, OATP1A5, OCT1, BCRP, MATE1, MATE2-K	[3, 7-9]<br>*[10]<br>**[11]
Norfloxacin (NOR)	400 mg p.o.	15	30-50	1.7	17.1 (renal)*	Metabolism (30%); renal (30%); biliary (30%)	3.0	OCT1, BCRP, MATE1, MATE2-K	[3, 9, 11, 12]
Ofloxacin (OFLX)	400 mg p.o.	25	80-99	2.4-3.5	15-21	Renal (80%); metabolism (< 5%); biliary (< 10%, unchanged)	5.0-7.5	OCT1, BCRP	[3, 9, 10, 13, 14]
**Third generation**									
Levofloxacin (LEV)	500 mg p.o.	24-38	99 - 100	1.1-1.5	8.8 ± 1.7	Renal (> 87%); limited metabolism	4.0-7.0	OATP and possibly P-gp<br><br>OATP1A2, OCT1	[3, 9, 11, 15]
Pazufloxacin (PZFX)	300 mg i.v. 1h infusion<br><br>500 mg i.v.0.5h infusion	NA	NA	 <br>00.62 ± 0.11	21.7 ± 3.5<br><br>13.26 ± 4.55	Renal (72.3 ± 20.8%)	2.99 ± 0.5	Possibly P-gp and MATE1*	[16, 17]
Tosufloxacin (TFLX)	204 mg p.o.	~ 37%		143 (Vd/F)	12.12 ± 0.6	Renal (35.4 ± 2.2 %)	3.49 ± 0.23		[18, 19]
**Fourth generation**									
Moxifloxacin (MXF)	400 mg	30-50	86 - 90	1.7-2.7	12 ± 2	Metabolism; renal (20%); biliary (25%)	11-15.5 h	OCT1	[3, 7, 9, 11, 20]
Lascufloxacin (LSFX)	100 mg p.o. - 100 mg i.v.	74	-	2.46 – 4.11	7.62 - 8.07		15.6 - 18.2	MDR1, BCRP	[21-23]

Sitafloxacin (STFX)	50 - 500 mg p.o. and 500 mg i.v.	50	89	1.46 – 2.57	15.8-19.7	Renal (~70%, filtration and tubular secretion), metabolism	5.19 - 7.0		[24-27]

References

[1] T.B. Vree, W.J. Wijnands, A.M. Baars, Y.A. Hekster, Pharmacokinetics of nalidixic acid in man: hydroxylation and glucuronidation, Pharmaceutisch Weekblad. Scientific Edition, 10 (1988) 193-199.

[2] G. Barbeau, P.M. Belanger, Pharmacokinetics of nalidixic acid in old and young volunteers, Journal of Clinical Pharmacology, 22 (1982).

[3] P. Balasundaram, S. Veerappapillai, R. Karuppasamy, Quinolones and Fluoroquinolones to Treat Salmonella Typhimurium: A Review of Metabolism and Pharmacokinetics, Current Drug Metabolism, 18 (2017) 1085-1094.

[4] N.F.G. Cuisinaud, N. Pozet, P.Y. Zech, J. Sassard, Nalidixic acid kinetics after single and repeated oral doses, Clinical Pharmacology and Therapeutics, 29 (1981) 695-698.

[5] T. Izumi, T. Kitagawa, Protein binding of quinolonecarboxylic acids. I. Cinoxacin, nalidixic acid and pipemidic acid, Chemical and Pharmaceutical Bulletin, 37 (1989) 742-745.

[6] B.C. Burckhardt, M. Henjakovic, Y. Hagos, G. Burckhardt, Differential interaction of dantrolene, glafenine, nalidixic acid, and prazosin with human organic anion transporters 1 and 3, The Journal of Pharmacology and Experimental Therapeutics, 362 (2017) 450-458.

[7] B. Viaggi, A. Cangialosi, M. Langer, C. Olivieri, A. Gori, A. Corona, S. Finazzi, A. Di Paolo, Tissue Penetration of Antimicrobials in Intensive Care Unit Patients: A Systematic Review—Part II, Antibiotics, 11 (2022) 1193.

[8] A. Shah, M.C. Liu, D. Vaughan, A.H. Heller, Oral bioequivalence of three ciprofloxacin formulations following single-dose administration: 500 mg tablet compared with 500 mg/10 mL or 500 mg/5 mL suspension and the effect of food on the absorption of ciprofloxacin oral suspension, Journal of Antimicrobial Chemotherapy, 43 Suppl A (1999) 49-54.

[9] K. Lin, R. Wang, T. Li, Y. Zuo, S. Yang, D. Dong, Y. Zhu, Drug Transporters and Metabolizing Enzymes in Antimicrobial Drug Pharmacokinetics: Mechanisms, Drug–Drug Interactions, and Clinical Implications, Biomolecules, 15 (2025) 864.

[10] H. Lode, G. Höffken, P. Olschewski, B. Sievers, A. Kirch, K. Borner, P. Koeppe, Pharmacokinetics of ofloxacin after parenteral and oral administration, Antimicrobial Agents and Chemotherapy, 31 (1987) 1338-1342.

[11] A. Wiesner, P. Zagrodzki, A. Gawalska, P. Paśko, Together or Apart? Revealing the Impact of Dietary Interventions on Bioavailability of Quinolones: A Systematic Review with Meta-analyses, Clinical Pharmacokinetics, 63 (2024) 773-818.

[12] B.N. Swanson, V.K. Boppana, P.H. Vlasses, H.H. Rotmensch, R. Ferguson, Norfloxacin disposition after sequentially increasing oral doses, Antimicrobial Agents and Chemotherapy, 23 (1983) 284-288.

[13] D.E. Martin, J. Shen, J. Griener, R. Raasch, J.H. Patterson, W. Cascio, Effects of ofloxacin on the pharmacokinetics and pharmacodynamics of procainamide, Journal of Clinical Pharmacology, 36 (1996) 85-91.

[14] D. Guay, K. Tack, S. Flor, Safety and pharmacokinetics of single dose intravenous ofloxacin in healthy volunteers, International Journal of Clinical Pharmacology Research, 11 (1991) 203-209.

[15] A.W. Wallace, J.M. Victory, G.W. Amsden, Lack of bioequivalence when levofloxacin and calcium-fortified orange juice are coadministered to healthy volunteers, Journal of Clinical Pharmacology, 43 (2003) 539-544.

[16] J. Lee, S.J. Seong, M.S. Lim, S.M. Park, J. Park, J.J. Seo, H.W. Lee, Y.R. Yoon, Single-dose pharmacokinetics and dose proportionality of intravenous pazufloxacin mesilate in healthy Korean volunteers, Expert Opinion on Drug Metabolism & Toxicology, 8 (2012) 921-928.

[17] S. Uegami, K. Ikawa, H. Ohge, A. Nakashima, N. Shigemoto, N. Morikawa, Y. Murakami, T. Sueda, Pharmacokinetics and pharmacodynamic target attainment of intravenous pazufloxacin in the bile of patients undergoing biliary pancreatic surgery, Journal of Chemotherapy, 26 (2014) 287-292.

[18] R. Minami, N. Inotsume, C. Nakamura, M. Nakano, Stereoselective analysis of the disposition of tosufloxacin enantiomers in man, European Journal of Clinical Pharmacology, 45 (1993) 489-491.

[19] Y. Niki, Pharmacokinetics and safety assessment of tosufloxacin tosilate, Journal of Infection and Chemotherapy, 8 (2002) 1-18.

[20] C.M. Culley, M.K. Lacy, N. Klutman, B. Edwards, Moxifloxacin: clinical efficacy and safety, American Journal of Health-System Pharmacy, 58 (2001) 379-388.

[21] H. Furuie, S. Tanioka, K. Shimizu, S. Manita, M. Nishimura, H. Yoshida, Intrapulmonary Pharmacokinetics of Lascufloxacin in Healthy Adult Volunteers, Antimicrobial Agents and Chemotherapy, 62 (2018) e02169-02117.

[22] K. Ohya, J. Takano, S. Manita, In Vitro Mechanistic Study of the Distribution of Lascufloxacin into Epithelial Lining Fluid, Antimicrobial Agents and Chemotherapy, 63 (2019) e02208-02218.

[23] H. Furuie, S. Tanioka, K. Shimizu, S. Manita, M. Nishimura, H. Yoshida, Intrapulmonary pharmacokinetics of lascufloxacin in healthy adult volunteers, Antimicrobial agents and chemotherapy, 62 (2018) 10.1128/aac. 02169-02117.

[24] G. Wu, L. Wu, X. Hu, H. Zhou, J. Liu, M. Zhu, Y. Zheng, Y. Zhai, J. Shentu, Pharmacokinetics and safety of sitafloxacin after single oral doses in healthy volunteers, International Journal of Clinical Pharmacology and Therapeutics, 52 (2014) 1037-1044.

[25] Y. Lei, P. Luo, Y. Chen, L. Xue, D. Zhang, X. Qi, Y. Fang, H. Yu, Z. He, Q. Zuo, C. Liu, D. Liu, X. Ren, Q. Chen, Pharmacokinetics and Safety Evaluation of a New Generic Sitafloxacin: A Phase I Bioequivalence Study in Healthy Chinese Participants, Clinical Pharmacology in Drug Development, 14 (2025) 592-597.

[26] M. Nakashima, T. Uematsu, K. Kosuge, K. Umemura, H. Hakusui, M. Tanaka, Pharmacokinetics and tolerance of DU-6859a, a new fluoroquinolone, after single and multiple oral doses in healthy volunteers, Antimicrobial Agents and Chemotherapy, 39 (1995) 170-174.

[27] J. O'Grady, A. Briggs, S. Atarashi, H. Kobayashi, R.L. Smith, J. Ward, C. Ward, D. Milatovic, Pharmacokinetics and absolute bioavailability of sitafloxacin, a new fluoroquinolone antibiotic, in healthy male and female Caucasian subjects, Xenobiotica, 31 (2001) 811-822.
