# Supplementary material for: Quinolone Antimicrobials: A Medicinal Chemistry Perspective on Structural Evolution, Pharmacology, and Computational Profiling
Source: Molecules. 2026 Nov 2;31(21):3949. doi: 10.3390/molecules31213949 (PMC13765324; doi:10.3390/molecules31213949)
Supplement: Supplementary file 1 [file molecules-31-03949-s001.zip › Supplementary Table S5.pdf]

Supplementary Table S5. Tissue penetration and PK/PD index of some quinolones.

Quinolone/PK	Tissue Penetration *(PRu)*	PK/PD Index	References
Ciprofloxacin	Muscle – 1.23 ± 0.24<br>Subcutaneous tissue 0.89 ± 0.16	Primary:<br>$fAUC_{0-24}$/MIC ≥ 90-100 (Gram-negative bacteria)<br><br>$fAUC_{0-24}$/MIC = 30-50 (Gram-positive bacteria)<br><br>Secondary (prevention of resistance):<br>C_{max}/MIC ≥ 8-10	[1-3]
Gemifloxacin	Muscle – 1.7 ± 0.7<br>Adipose tissue – 2.4 ± 1.0	$fAUC_{0-24}$/MIC around 30 based on results from in vitro and animal models of infection<br><br>$fAUC_{0-24}/MIC_{90}$ 99.3–132.3 *(S. pneumoniae* in healthy subjects after multiple doses)	[4]
Levofloxacin	Adipose tissue:<br>1.1 ± 0.6 healthy<br>1.2 ± 1.0 inflamed	$fAUC_{0-24}$/MIC ≥ 87-100 (Gram-negative bacteria)<br><br>$fAUC_{0-24}$/MIC ≥ 30 *S. pneumoniae* in the lungs	[5-7]
Moxifloxacin	Muscle – 0.86 ± 0.17<br>Subcutaneous tissue 0.81 ± 0.19<br><br>Subcutaneous adipose tissue*:<br>Non-diabetic patient:<br>Healthy tissue – 0.5 ± 0.3<br>Inflamed tissue – 1.2 ± 0.8<br>Diabetic patient:<br>Healthy tissue - 0.9 ± 0.6<br>Inflamed tissue – 0.5 ± 0.4	Primary:<br>$fAUC_{0-24}$/MIC = 100 - 125 (Gram-negative bacteria)<br><br>$fAUC_{0-24}$/MIC = 30 - 50 (Gram-positive bacteria)<br><br>Secondary (prevention of resistance)<br>C_{max}/MIC ≥12.2<br><br>$fAUC_{0-24}$/MIC >192 for *S. pneumoniae*<br>C_{max}/MIC = 18 for *S. pneumoniae*<br>C_{max}/MIC ≥ 75 for *H. influenzae* and *M. catarrhalis*.	[8-11]
Ofloxacin	Subcutaneous tissue – 0.716 ± 0.185	$fAUC_{0-24}$/MIC > 100 – 125	[9, 12, 13]

Pazufloxacin		Primary<br>$f\mathrm{AUC}_{0-24}$/MIC > 100.8<br><br>Secondary<br>fC_{max}/MIC = 10.8	[14]
Tosufloxacin		$f\mathrm{AUC}_{0-24}$/MIC 138 and 193 for *S. pneumoniae*<br><br>Secondary<br>fC_{max}/MIC > 10 *(S. pneumoniae)*	[15]

PRu = Tissue penetration ratio (AUC of ISFu to AUC of unbound plasma concentration)
ISFu = unbound interstitial fluid concentrations
*f*AUC = area under the unbound plasma concentration curve
*f*Cmax – unbound peak concentration in plasma
*No statistical differences among all groups

References

[1] M. Brunner, U. Hollenstein, S. Delacher, D. Jäger, R. Schmid, E. Lackner, A. Georgopoulos, H.G. Eichler, M. Müller, Distribution and antimicrobial activity of ciprofloxacin in human soft tissues, Antimicrobial Agents and Chemotherapy, 43 (1999) 1307-1309.

[2] M.G. Papich, Pharmacokinetic-pharmacodynamic (PK-PD) modeling and the rational selection of dosage regimes for the prudent use of antimicrobial drugs, Veterinary Microbiology, 171 (2014) 480-486.

[3] N.J. Onufrak, A. Forrest, D. Gonzalez, Pharmacokinetic and Pharmacodynamic Principles of Anti-infective Dosing, Clinical Therapeutics, 38 (2016) 1930-1947.

[4] S.M. Bhavnani, D.R. Andes, Gemifloxacin for the treatment of respiratory tract infections: in vitro susceptibility, pharmacokinetics and pharmacodynamics, clinical efficacy, and safety, Pharmacotherapy, 25 (2005) 717-740.

[5] R. Bellmann, G. Kuchling, P. Dehghanyar, M. Zeitlinger, E. Minar, B.X. Mayer, M. Müller, C. Joukhadar, Tissue pharmacokinetics of levofloxacin in human soft tissue infections, British Journal of Clinical Pharmacology, 57 (2004) 563-568.

[6] H.Y. Huang, C.F. Wang, P.L. Lu, S.P. Tseng, Y.L. Wang, T.C. Chen, K. Chang, H.P. Tu, S.Y. Lin, Clinical Impact of the Revised 2019 CLSI Levofloxacin Breakpoints in Patients with Enterobacterales Bacteremia, Antimicrobial Agents and Chemotherapy, 65 (2021) e00074-00021.

[7] A.M. Noreddin, T.K. Marras, K. Sanders, C.K. Chan, D.J. Hoban, G.G. Zhanel, Pharmacodynamic target attainment analysis against Streptococcus pneumoniae using levofloxacin 500 mg, 750 mg and 1000 mg once daily in plasma (P) and epithelial lining fluid (ELF) of hospitalized patients with community acquired pneumonia (CAP), International Journal of Antimicrobial Agents, 24 (2004) 479-484.

[8] M. Müller, H. Stass, M. Brunner, J.G. Möller, E. Lackner, H.G. Eichler, Penetration of moxifloxacin into peripheral compartments in humans, Antimicrobial Agents and Chemotherapy, 43 (1999) 2345-2349.

[9] S.P. Zvada, P. Denti, F.A. Sirgel, E. Chigutsa, M. Hatherill, S. Charalambous, S. Mungofa, L. Wiesner, U.S. Simonsson, A. Jindani, T. Harrison, H.M. McIlleron, Moxifloxacin population pharmacokinetics and model-based comparison of efficacy between moxifloxacin and ofloxacin in African patients, Antimicrobial Agents and Chemotherapy, 58 (2014) 503-510.

[10] C. Joukhadar, H. Stass, U. Müller-Zellenberg, E. Lackner, F. Kovar, E. Minar, M. Müller, Penetration of moxifloxacin into healthy and inflamed subcutaneous adipose tissues in humans, Antimicrobial Agents and Chemotherapy, 47 (2003) 3099-3103.

[11] K. Öbrink-Hansen, T.F. Hardlei, B. Brock, S. Jensen-Fangel, M. Kragh Thomsen, E. Petersen, M. Kreilgaard, Moxifloxacin pharmacokinetic profile and efficacy evaluation in empiric treatment of community-acquired pneumonia, Antimicrobial Agents and Chemotherapy, 59 (2015) 2398-2404.

[12] S. Bielecka-Grzela, A. Klimowicz, Evaluation of ofloxacin penetration into the skin after a single oral dose assessed by cutaneous microdialysis, Polish Journal of Pharmacology, 55 (2003) 613-618.

[13] K.J. Madaras-Kelly, B.E. Ostergaard, L.B. Hovde, J.C. Rotschafer, Twenty-four hour area under the concentration-time curve/MIC ratio as a generic predictor of fluoroquinolone antimicrobial effect using three strains of Pseudomonas aeruginosa and an in vitro pharmacodynamic model, Antimicrobial Agents and Chemotherapy, 40 (1996) 627-632.

[14] Y. Umezaki, K. Matsumoto, K. Ikawa, Y. Yokoyama, Y. Enoki, A. Shigemi, E. Watanabe, K. Nakamura, K. Ueno, H. Terazono, N. Morikawa, Y. Takeda, Concentration-Dependent Activity of Pazufloxacin against Pseudomonas aeruginosa: An In Vivo Pharmacokinetic/Pharmacodynamic Study, Antibiotics, 11 (2022) 982.

[15] Y. Fukuda, M. Takahata, J. Mitsuyama, Pharmacodynamic evaluation of tosufloxacin against Streptococcus pneumoniae in an in vitro model simulating serum concentration, Journal of Infection and Chemotherapy, 12 (2006) 1-8.
